# Iron in leaves: chemical forms, signalling, and in-cell distribution

**DOI:** 10.1093/jxb/erac030

**Published:** 2022-02-01

**Authors:** Máté Sági-Kazár, Katalin Solymosi, Ádám Solti

**Affiliations:** 1 Department of Plant Physiology and Molecular Plant Biology, Institute of Biology, ELTE Eötvös Loránd University, Pázmány Péter sétány 1/C, Budapest, H-1117, Hungary; 2 Doctoral School of Biology, Institute of Biology, ELTE Eötvös Loránd University, Pázmány Péter sétány 1/C, Budapest, H-1117, Hungary; 3 Department of Plant Anatomy, Institute of Biology, ELTE Eötvös Loránd University, Pázmány Péter sétány 1/C, Budapest, H-1117, Hungary; 4 Hasselt University, Belgium

**Keywords:** Chloroplast, citrate, DNA methylation, glutaredoxin, glutathione, hemerythrin, histone modification, iron–sulfur cluster, mesophyll, mitochondrion

## Abstract

Iron (Fe) is an essential transition metal. Based on its redox-active nature under biological conditions, various Fe compounds serve as cofactors in redox enzymes. In plants, the photosynthetic machinery has the highest demand for Fe. In consequence, the delivery and incorporation of Fe into cofactors of the photosynthetic apparatus is the focus of Fe metabolism in leaves. Disturbance of foliar Fe homeostasis leads to impaired biosynthesis of chlorophylls and composition of the photosynthetic machinery. Nevertheless, mitochondrial function also has a significant demand for Fe. The proper incorporation of Fe into proteins and cofactors as well as a balanced intracellular Fe status in leaf cells require the ability to sense Fe, but may also rely on indirect signals that report on the physiological processes connected to Fe homeostasis. Although multiple pieces of information have been gained on Fe signalling in roots, the regulation of Fe status in leaves has not yet been clarified in detail. In this review, we give an overview on current knowledge of foliar Fe homeostasis, from the chemical forms to the allocation and sensing of Fe in leaves.

## Teaser

Following iron transport to leaves, proper control over its location and incorporation is required, while sensing of the organellar iron status is also crucial.

## Introduction

Iron (Fe) is one of the most important transition metals in plants required for essential cell functions. The known oxidation states of Fe in biological environments range from Fe(II) to Fe(IV) (heme), the chemistry of which is multiplied by accessible spin states (for review, see Chen and Browne, 2018). In biological systems, Fe-dependent redox reactions occur in a broad electrochemical potential. In consequence, Fe cofactor-containing redox enzymes are common. In plants, the photosynthetic apparatus has a particularly high need for these Fe cofactors. Thus, foliar Fe homeostasis plays a crucial role in the physiological status of the plant, since autotrophy relies on the availability of Fe (Box 1).

## Mobile iron species in leaves

Whether to maintain function or prevent harmful effects, the coordination of transition metals in biological systems is always essential. Nevertheless, the coordination of transition metals and the stability of the complexes are influenced by several factors in a highly complex multiequilibrium system such as the biological environment. Thus, ligand concentration and availability, the local pH, the p*K*_a_ of the ligand(s), the presence of competing ligands, and the presence of metals other than Fe that also have the potential to form stable complexes together determine the occurrence of any Fe compounds in plants. Fe has a high affinity to, among others, oxygen (O), nitrogen (N), and sulfur (S). With its higher charge-to-size ratio, Fe(III) shows better affinity to negatively charged ligands and especially negatively charged O-ligands, while Fe(II) prefers aromatic N-ligands and S-ligands (Harris, 2002). In aqueous solutions, the deprotonation of the coordinating water molecules of Fe ions is prevalent for Fe(III) even at lower pH values (Hider and Kong, 2013). At neutral pH, Fe(III)-OH complexes crosslink into insoluble ferrihydrite polymers, while Fe(II) will remain soluble even at higher concentrations. Although Fe(II) is susceptible to autoxidation under oxygenic conditions, the reducing environment in the symplast favours maintenance of the reduced status of Fe. Since at neutral pH the concentration of free OH^–^ ions exceeds the threshold of Fe(III)-OH formation, the maintenance of the reducing environment and thus the provision of available Fe(II) for metabolic processes is essential. In consequence, in a biological system the varying pH conditions require multiple strategies for the coordination of Fe species.

O-ligands of various denticities generally possess lower p*K*_a_ values to Fe compared with N-ligands, implying their dominance at lower pH values (Table 1). In apoplastic spaces and acidic compartments of the cells, such as the xylem sap, the apoplast, the vacuole, and the intermembrane spaces of both chloroplasts and mitochondria, the low amount of free OH^–^ ions does not contest the formation of carboxyl complexes of Fe due to the high abundance of deprotonated carboxyl groups (Martell and Hancock, 1996). Among carboxyl O-ligands, citrate (Cit) and malate (Mal) are prevalent in these spaces. In accordance, Cit and Mal biosynthesis, and their concentrations in the apoplastic aqueous phases, constitutively increase under Fe deficiency (López-Millán *et al*., 2000; Larbi *et al.*, 2010; Sebastian and Prasad, 2018, Seregin and Kozhevnikova, 2020). Organic acid accumulation in the vacuoles also contributes to the complexing, and thus storage, of Fe under the acidic pH of the vacuoles. (Roschzttardtz *et al.*, 2009; Flis *et al.*, 2016). Cit and Mal may act as tri- and bidentate ligands, respectively, forming stable Fe-chelates, while at mixed ligand composition they also form stable polynuclear complexes (Silva *et al.*, 2009; Flis *et al.*, 2016). At low pH, the Cit complex of Fe(II) is stable, but under moderately acidic conditions it reoxidizes over time and forms stable polynuclear complexes of Fe(III). Thus, taking into account the pH of the apoplast spaces in plants, carboxyl ligands complex Fe(III). Moreover, the formation of stable polynuclear complexes following the reoxidation of Fe(II)-carboxylates would decrease the availability of Fe that is not favoured under biological conditions. Cit has a clear advantage for Fe complexation over Mal, as it requires a 1:2 stoichiometry to Fe. In comparison, the Mal concentration has to be at least three times that of Fe to achieve similar complex levels as with Cit (Monsant *et al.*, 2011) (Table 1). Moreover, the concentration of Cit exceeds that of Mal in acidic compartments. Therefore, the formation of Cit complexes of Fe(III) is favoured in plants. Nevertheless, in both the xylem sap and acidic environments of plant cells, amino acids that could compete for Fe are also abundant. As for O-ligands, the amino acids glutamate (Glu) and aspartate (Asp) are also important. Being bidentate Fe ligands, they can effectively compete with other carboxylates for Fe *in vitro* (Table 1) (Aravind and Prasad, 2005; Cui *et al.*, 2020). Nevertheless, as Cui *et al.* (2020) pointed out, based on the glutamate synthase mutant *glu1-4*, Glu complexes of Fe cannot be a major form, especially in the xylem. However, the role of Glu complexes of Fe in organism-level Fe signalling cannot be excluded, but a specific signal requires associated signal perception, too. Nevertheless, the concentration of Cit ensures the dominance of its complexes of Fe, especially in the xylem sap.

**Table 1.** Iron-ligand affinities for relevant complexes in foliar iron homeostasis

Ligand			p*K*_a_∗						Complex stoichiometry	Affinity constant (logK) for different stoichiometry†		pH range
Name	Type	Denticity	p*K*_a_1	p*K*_a_2	p*K*_a_3	p*K*_a_4	p*K*_a_5	p*K*_a_6		Fe(II)	Fe(III)	
Cit	O–	3	3.128^*a*^	4.761^*a*^	6.396^*a*^	–	–	–	1:1	4.4^*h*^	11.5^*h*^	5.5–6^*n*^
									1:2	n.d.	32.73^*i*^	
Mal	O–	2	3.4^*b*^	5.11^*b*^	–	–	–	–	1:1	2.6^*h*^	7.1^*h*^	n.d.
									1:3	n.d.	n.d.	
Asp	N– O–	3	1.99^*b*^	3.9^*b*^	9.9^*b*^	–	–	–	1:1	5.34^*j*^	11.4^*j*^	n.d.
									1:2	8.57^*j*^	n.d.	
Cys	N– O– S–	2 (3)	1.71^*a*^	8.36^*a*^	10.75^*a*^	–	–	–	1:1	6.69^*j*^	11.9^*j*^	~8^*d*^
									1:2	11.9^*j*^	14.49^*j*^	
His	N– O–	3	1.5^*a*^	6.07^*a*^	9.34^*a*^	–	–	–	1:1	5.8^*j*^^,^‡	4.4^*j*^^,^‡	6–8^*o*^
									1:2	10.43^*j*^^,^‡	n.d.	
Glu	N– O–	3	2.19^*c*^	4.25^*c*^	9.67^*c*^	–	–	–	1:1	3.3^*j*^^,^‡	13.7^*j*^^,^‡/11.8^*k*^	5–n.d.^*j*^
									1:2	n.d.	n.d.	
GSH	S–	1	2.12^*b*^	3.52^*b*^	8.67^*b*^	9.57^*b*^,^*d*^	–	–	1:1	5.12^*d*^	n.d.	6.5–8^*d*^
NA	N– O–	6	<1.5^*e*^	2.35^*e*^	2.86^*f*^	6.92^*f*^	9.14^*f*^	10.09^*f*^	1:1	12.8^*l*^	20.6^*f*^	6–8^*f*^
H_2_O/OH^–^	O–	1	14^*g*^	–	–	–	–	–	1:1	3.6^*m*^	11.81^*m*^	–

Ionic strength μ=0–0.1 M, at room temperature unless otherwise stated, except glutamate and nicotianamine, where the original publication provided no data.

Ionic strength μ=0–0.15 M, at room temperature.

Value is corrected according to Martell and Hancock (1996), due to different ionic strength.

References are identified with letters:

^*a*^ Goldberg *et al.*, 2002;

^*b*^ Lide, 2004;

^*c*^ O’Neil, 2013;

^*d*^ Hider and Kong, 2011;

^*e*^ Hider *et al.*, 2004;

^*f*^ von Wirén *et al*., 1999;

^*g*^ Silverstein and Heller, 2017;

^*h*^ Martell and Smith, 1977;

^*i*^ Silva *et al.*, 2009;

^*j*^ Murphy *et al.*, 2020;

^*k*^ Prasetyo *et al.*, 2020;

^*l*^ Anderegg and Ripperger, 1989;

^*m*^ Martell and Hancock, 1996;

^*n*^ Hanikenne *et al.*, 2021;

^*o*^ Hider *et al.*, 2021.

Asp, aspartate; Cit, citrate; Cys, cysteine; Glu, glutamate; GSH, glutathione; His, histidine; Mal, malate; NA, nicotianamine; n.d., not detectable.

At close to neutral pH, the increasing competition of carboxylates with free OH^–^ ions resulted in enhanced Fe(III)-OH formation and thus Fe precipitation (Martell and Hancock, 1996; Hider and Kong, 2010). In consequence, the coordination of Fe with O-ligands is not favoured in the phloem sap, the cytosol, the matrix of mitochondria, and the stroma of chloroplasts. Indeed, amino acids, particularly histidine (His) and cysteine (Cys), are considered to be effective ligands of Fe under these conditions (Aravind and Prasad, 2005; Seregin and Kozhevnikova, 2020). Both His and Cys have high affinity to Fe (Table 1) and are able to maintain the reduced state of Fe(II) at neutral pH. Together with other thiol-containing compounds, the S-ligand of Cys has a primary importance to maintain the reduced status of Fe under symplastic conditions (Bhattacharyya et al., 2013; 2019; Murphy *et al.*, 2020). For instance, Cys is a major component in the low-molecular-weight thiol glutathione (GSH). GSH has a high capacity to reduce Fe(III) and serve as a ligand for Fe(II) (Martell and Hancock, 1996; Hider and Kong, 2011). At cytoplasmic pH and concentration, GSH forms complexes with Fe(II) of higher stability than those with Cit (Table 1). Since the cytoplasmic concentration of GSH, in the low mM range, exceeds the concentration of free amino acids (Meyer *et al.*, 2001; Hider and Kong, 2011; Hider *et al.*, 2021), GSH is supposedly the major Fe(II) ligand in the cytoplasm. Moreover, Fe(II)-GSH takes part in Fe–S biosynthesis and in the formation of nitrosyl-Fe complexes. As well as forming peptides such as GSH, amino acids are also important to build proteins. Although amino acid residues are also important ligands of Fe, especially under symplastic conditions (Balk and Pilon, 2011; Balk and Schaedler, 2014), the protein-bound Fe pool is not readily mobilized and thus not significant in the delivery of Fe. One of the few examples might be IRON MAN 1, a long-distance Fe signalling peptide that contains a stretch of Asp residues with the potential affinity to bind and deliver Fe (Grillet *et al.*, 2018).

Along with His, Cys, and GSH, nicotianamine (NA) is an N-ligand compound that is a key component in metal homeostasis of higher plants. NA is derived from methionine through the condensation of three *S*-adenosylmethionine molecules by NA synthase (Curie *et al.*, 2009; Clemens *et al.*, 2013). It has very high affinity to Fe as a hexadentate ligand, forming stable Fe chelates (Table 1). At quasi-neutral pH, Fe-NA complexes are kinetically stable, while at acidic pH total ligand exchange occurs with Cit within 5 min (von Wirén *et al*., 1999). Removal of cytosolic NA results in impaired intracellular Fe movement in leaves and leads to symptoms of Fe deficiency (Haydon *et al.*, 2012; Lee *et al.*, 2021). Information on the abundance of NA in the symplast is, however, not available. In consequence, it is not clear whether any potential ligands forming Fe complexes with high stability are present in the symplast in a concentration high enough to compete with NA for Fe. Indeed, the lack of information on the intracellular NA concentration suggests that the intracellular NA concentration may be significantly lower than that of GSH. Therefore, it seems that NA supports the intracellular mobility of Fe rather than forming a significant pool of Fe complexes. In NA-deficient *chloronerva* mutants of tomato, Fe phosphate deposits appear (Becker *et al.*, 1995; Liu *et al.*, 1998). Thus, NA is probably involved in keeping liberated Fe soluble (Curie and Briat, 2003). Since no signal of either Fe(II)-NA or Fe(III)-NA can be detected in mature chloroplasts based on Mössbauer spectroscopic analysis (Solti *et al.*, 2012; Müller *et al.*, 2019), the function of Fe-NA complexes seems to be restricted to coping with liberated Fe, but they do not form a major pool. NA and GSH supposedly act as ligands for Fe under different conditions in the symplast, where GSH could be at least in part responsible for multiple aspects of the labile Fe pool, whereas NA might be involved in retaining Fe solubility and in the support of transmembrane Fe transport. Nevertheless, ligand exchange between NA and GSH has not yet been revealed.

## Iron transport in leaves

**Fig. 1.**
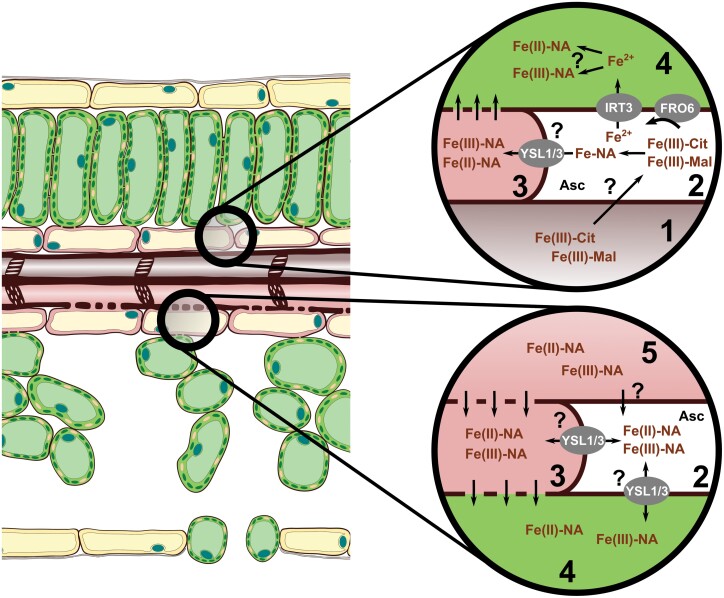
Fe transport processes in leaves. Leaves of dicot plants such as Arabidopsis primarily receive Fe through the vasculature via root-to-shoot Fe translocation in the xylem vessels or from source–sink Fe redistribution processes operated by the phloem. Fe(III)-Cit is the dominant species of Fe in the xylem (1) that reaches the leaf tissues. Fe-carboxylates originating from the xylem sap are thought to infiltrate the intracellular spaces and the apoplast (2). Although under the slightly acidic environment in the apoplastic spaces Fe(III)-carboxylates have a high stability, ligand exchange towards NA has not been clarified yet. Parenchyma cells around the vasculature (3) express YSL1 and YSL3, thus Fe-NA uptake is also suggested. Mesophyll cells (4) primarily operate a reduction-based method of Fe acquisition. FRO family enzymes can utilize Fe(III)-carboxylate complexes; Asc-mediated reduction and photoreduction of Fe(III)-carboxylates might be also involved, as shown by the redundant nature of the ferric chelate reductase activity of the mesophyll. In addition to Fe translocation in the xylem, the phloem (5) is also involved in the redistribution and transport of Fe, although the magnitude of xylem transportation is supposedly significantly higher. In all cytoplasm-filled environments, including the phloem, the presence of Fe-NA species is suggested to keep Fe soluble. Moreover, the transport of Fe species involved in long-distance Fe signalling may also use the phloem transportation pathway. The nature of phloem unloading with respect to Fe species has not been clarified yet. Cells that are symplastically connected through plasmodesmata might not need special Fe transporters. Indeed, the presence of YSL transporters, especially during leaf development and at senescence initiation, indicate that the transport of Fe-NA species is also important in the phloem transport of Fe. For abbreviations and further details see the text.

**Fig. 2.**
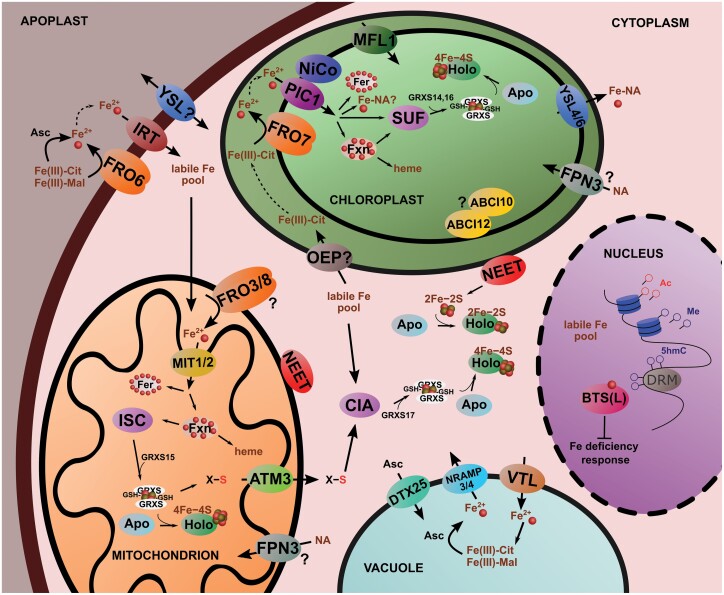
Fe homeostasis of mesophyll cells. Mesophyll cells of dicots such as Arabidopsis primarily operate a reduction-based Fe acquisition mechanism, whereas the extent of YSL-mediated Fe uptake is less well characterized. IRT-type transporters are suggested to mediate the transport of divalent metals. Although transmembrane transport is generally accepted to be based on the free forms, Fe should be complexed in the cytoplasm to avoid ROS generation. Fe complexed by low-molecular-weight ligands is thought to be part of the labile Fe pool of the cells. The proper composition of this labile Fe pool is yet to be understood. FRO family enzymes are targeted into both chloroplasts and mitochondria, thus their Fe acquisition is supposedly dominated by the reduction-based pathway, too. Fe import into the mitochondrial matrix and chloroplast stroma is a complex process because of the double-envelope system of the organelles. Nevertheless, functional characterization of the protein members of this machinery is far from complete, as discussed in Vigani *et al.* (2019). In Fe acquisition by chloroplasts, PIC1, NiCo, and MFL components can cooperate, whereas mitochondria may primarily operate MIT1 and MIT2. Fe in the chloroplasts and mitochondria is directed towards incorporation into hemes and Fe–S clusters. Hemes are synthesized by ferrochelatases, but frataxin is considered to be involved in heme biosynthesis in mitochondria. Mitochondria and chloroplasts operate the ISC and SUF systems, respectively, for the biogenesis of Fe–S clusters. Plant GRXs are involved in the management and insertion of Fe–S clusters into apoproteins. The export of Fe–S clusters towards the cytoplasm involves NEET proteins and ABC transporters. Both the photosynthetic and respiratory electron transport chains require a significant amount of Fe, thus in mesophyll cells, Fe is primarily directed towards protein complexes operating these processes. During the decomposition of these systems, Fe can be liberated. YSL family transporters are considered to be involved in retaining the solubility of Fe and exporting Fe out of the plastids. In addition to the organellar Fe–S cluster biosynthesis, in the cytoplasm the eukaryotic CIA system, which partly depends on the mitochondrial ISC system as a source of reduced sulfur, is operational. Although the vacuoles of the leaf cells contribute to the temporal storage of Fe, the primary Fe storage that helps to manage temporal Fe excess or Fe liberation are ferritins (also illustrated in Fig. 3). Since Fe is a potentially toxic element, proper control over cellular Fe status is essential. Hemerythrin domain proteins BTS(L) were described as Fe sensors of plant cells. Nevertheless, the complexity of Fe-status-connected responses in plant cells suggests the existence of multiple sensing and regulatory mechanisms, mediated by small molecules and Fe–S sensing mechanisms. Although the understanding of epigenetic mechanisms that regulate cellular Fe homeostasis is far from complete, both DNA methylation by Domains Rearranged Methyltransferases (DRM) and histone modifications are important signals. For abbreviations and further details see the text.

Leaves primarily receive Fe originating from root Fe uptake. In the xylem, Fe is transported predominantly as Fe(III)-carboxylates (Fig. 1) such as Fe(III)_3_-(Cit)_3_ (Rellán-Álvarez *et al.*, 2010). Impaired Cit loading into the xylem by Ferric Reductase Defective (FRD) 3 leads to Fe-deficiency symptoms in the shoot (Durrett *et al.*, 2007; Roschzttardtz *et al.*, 2011). Supplying Glu to *frd3* mutant Arabidopsis (*Arabidopsis thaliana*) plants restored leaf chlorosis (Cui *et al.*, 2020), suggesting a role of FRD3 in root-to-shoot Fe transport. Fe translocation towards the youngest leaves is suggested to be based on the sink–source Fe distribution through the phloem. With high enough concentrations in the phloem sap, NA is suggested to be the major ligand in the phloem-based Fe transport towards young leaves and reproductive organs, that is, tissues that are not reached by differentiated xylem vessels (Klatte *et al.*, 2009; Schuler *et al.*, 2012). In Arabidopsis leaves, Yellow Stripe-Like (YSL) 1 and YSL3 (Fig. 1) supposedly transport Fe-NA complexes from veins to surrounding parenchyma cells (Kumar *et al.*, 2017). However, *YSL1* and *YSL3* are expressed only in parenchyma cells and especially in senescing and cauline leaves (Waters *et al.*, 2006), hence we suggest they cannot be responsible for the complete Fe distribution within the whole leaf lamina and may instead function in Fe redistribution. The direction of the Fe transport mediated by YSLs has not been clarified yet. Since plant cells are symplastically connected via plasmodesmata, cell-to-cell Fe movement does not seem to require transporters. Additionally, in acidic compartments, ligand exchange between NA and carboxylates is likely (von Wirén *et al*., 1999); thus, the functional characterization and contribution of the foliar Fe transport of YSL1 and YSL3 requires further studies. *ysl1ysl3* double mutants fail to induce Fe-deficiency responses in the roots; thus, YSL1 and YSL3 were also proposed to be involved in long-distance Fe status signalling (Kumar *et al.*, 2017). The xylem sap infiltrates the apoplast, where Fe(III)-carboxylates are still the dominant Fe species (Fig. 1). Leaf cells operate a reduction-based Fe uptake utilizing both Fe(III)-Cit and Fe(III)-Mal (Fig. 1 and 2; Brüggemann *et al.*, 1993; Larbi *et al.*, 2001). In Arabidopsis, FRO6 targets the plasma membrane of mesophyll cells (Jeong *et al.*, 2008). Overexpression of *AtFRO6* in tobacco (*Nicotiana tabacum*) leads to increased tolerance of leaf Fe chlorosis (Li *et al.*, 2011). Ascorbate (Asc)-mediated reduction of Fe(III) was suggested as an obligatory step in Arabidopsis embryos prior to Fe uptake (Grillet *et al.*, 2014). Since Asc is present in the apoplast of vascular parenchyma facing xylem vessels in the leaves (Zechmann *et al.*, 2011), xylem unloading and Fe acquisition by the vascular parenchyma cells may rely in part on Asc-mediated reduction (Figs 1, 2). Nevertheless, in leaves as in aerial organs, the photoreduction of Fe could be an important factor that contributes to Fe acquisition. The contribution of light-irradiance-induced reduction to the Fe uptake into mesophyll cells has not been characterized yet. Once Fe is reduced, Iron Regulated Transporter (IRT) 1 and IRT2 have a primary role in transporting Fe(II) into the cytoplasm (Figs 1, 2). His residues on the cytosolic side of IRT1 are suggested to bind Fe, affecting its function (Cointry and Vert, 2019). In contrast to the situation in the roots, the expression of IRT1 is not enhanced by Fe deficiency in the leaves, which indicates a rather stable uptake by leaf cells (Bauer *et al.*, 2004). Although there is clear evidence that Fe-NA transporters are also involved in the distribution and redistribution of Fe, the functional characterization of this transport is still elusive.

## Iron in cofactors in leaves

Mesophyll cells take up a significant amount of Fe, where it is directed towards Fe-containing cofactors of redox enzymes. Thus, the largest proportion of Fe in leaves is incorporated into tetrapyrrole and Fe–S cofactors. Tetrapyrroles are crucial in all cell compartments for various electron transfer reactions such as respiratory and photosynthetic electron transport chains (Brzezowski *et al.*, 2015). Sirohemes, synthesized on the first branch of the tetrapyrrole pathway, are harboured by plastidial nitrite and sulfite reductases (Tripathy *et al.*, 2010; Askenasy and Stroupe, 2020). Hemes are synthesized from protoporphyrin IX with the insertion of Fe(II) by ferrochelatase (Solymosi and Myśliwa-Kurdziel, 2021). The direct mechanism of Fe donation during heme biosynthesis is unknown, although frataxin has been suggested to play a role in it (Fig. 2) (Maliandi *et al.*, 2011; Gomez-Casati *et al.*, 2018; Armas *et al.*, 2019). Free hemes are stable, thus they can contribute to the formation of the Fe pool in cells (Shviro and Shaklai, 1987; Sahini *et al.*, 1996; Hanna *et al.*, 2016). In the cytosol, hemes form 1:1 complexes with GSH (Hider and Kong, 2011; O’Keeffe *et al.*, 2021). Moreover, plastidial, cytosolic, and nuclear proteins have been also identified as heme-binding proteins (Shimizu *et al.*, 2020; Sylvestre-Gonon *et al.*, 2020). Since the ^57^Fe Mössbauer spectroscopy characteristics of hemes and 4Fe–4S clusters are hardly distinguishable, direct determination of the proportion of Fe incorporated into hemes is limited.

Fe–S clusters represent more electronegative cofactors when compared with hemes. Rhombic 2Fe–2S and cubane 4Fe–4S clusters are common in various proteins (for review, see Przybyla-Toscano et al., 2018, 2021). In chloroplasts and mitochondria, the photosynthetic and respiratory electron transport chains, respectively, have the highest demand for Fe–S clusters. The majority of Fe in the chloroplasts can be identified as 4Fe–4S clusters (Solti *et al.* 2012). Fe–S clusters are sensitive to molecular oxygen and unstable in aqueous solutions, and thus their coordination through Cys or His residues is essential. In all eukaryotic cells, both the mitochondrial iron–sulfur cluster assembly (ISC) pathway and the eukaryotic cytosolic iron-sulfur assembly (CIA) pathway are operational, whereas plants also operate the sulfur mobilization (SUF) system in their plastids (Fig. 2). Indeed, the CIA pathway is heavily dependent on mitochondria (Balk and Pilon, 2011; Lu, 2018). Frataxin serves as an Fe donor for the mitochondrial ISC system (Fig. 2). In plants, lack of frataxin is lethal, while knockdown mutants show impaired activity of Fe–S-containing mitochondrial enzymes (Gomez-Casati *et al.*, 2018; Armas *et al.*, 2020). Turowski *et al.* (2015) first reported that plant frataxin is double-localized to the chloroplasts too, hypothetically donating ferrous Fe for the plastidial SUF system (Fig. 2). The biosynthesis of 4Fe–4S clusters in mitochondria depends on a reduction step in which Ferredoxin (FDX) 2 provides the reducing power. In this reductive fusion of 2Fe–2S clusters, FDX1 cannot replace FDX2 (Weiler *et al.*, 2020). GSH can bind 2Fe–2S clusters in cytoplasmic conditions. GSH depletion impairs cytosolic Fe–S protein maturation and increases the Fe levels in the mitochondria (Kumar *et al.*, 2011; Qi *et al.*, 2012). Defects in GSH biosynthesis lead to the down-regulation of the essential Fe homeostasis genes *Natural Resistance-Associated Macrophage Protein* (*NRAMP*) *3 and NRAMP4, Permease In Chloroplast* (*PIC*) *1*, *Ferritin* (*FER*) *1*, and *IRT1* in Arabidopsis (Shee *et al.*, 2021, Preprint).

Monothiol (Class II) GRXs with Fe–S cluster transferase activity are also important in delivering Fe–S clusters (Berndt and Lillig, 2017; Trnka *et al.*, 2020; Talib and Outten, 2021). In Arabidopsis, Class II GRXS17, GRXS15, and GRXS14/16 are located in the cytoplasm, mitochondria, and chloroplasts, respectively (Fig. 2). Plant GRXS15 can restore mitochondrial Fe homeostasis in yeast *grx5* mutants (Couturier *et al.*, 2015). However, information on yeast GRXs cannot be directly translated to plant models. Although yeast Class II GRXs have a central role in the sensing of Fe–S clusters in mitochondria (Mühlenhoff *et al.*, 2020), such a role of plant GRXs has not been confirmed so far. Knockout mutation of Arabidopsis mitochondrial GRXS15 is lethal due to defective embryo development, whereas the mutation of plastidial GRXs affects the sensitivity to oxidative stress (Balk and Pilon, 2011; Moseler *et al.*, 2015). The fact that mutation of plastidial GRXs is not lethal suggests that the integration of Fe–S clusters into the apoproteins of the photosynthetic apparatus might be an independent action. Plant GRXs can form heterodimers with BOLA proteins (GRXS14/BOLA1 in plastids and GRXS15/BOLA4 in mitochondria; Roret *et al.*, 2014; Rey *et al.*, 2019). Although in yeast GRX/BOLA heterodimers are involved in Fe sensing by affecting gene expression (Rey *et al.*, 2019), such a role has not been documented in plants so far. In yeast, Fe–S clusters are proposed to be exported from mitochondria in the form of [2Fe–2S](GS)_4_^2–^ by ATM1 (Li and Cowan, 2015). However, Fe–S clusters are not stable in aqueous solution under aerobic conditions. Thus, ATM1 most likely transports an S-containing compound (Lill and Freibert, 2020). Regarding plant ATM3, Schaedler *et al.* (2014) proposed and Marty *et al.* (2019) confirmed that plant mitochondrial ATM3 exports GSH and persulfide-containing glutathione polysulfide, thus affecting cytoplasmic Fe–S cluster biosynthesis (Fig. 2). Moreover, ATM3 is indirectly involved in cyclic pyranopterin monophosphate (Moco cofactor intermediate) biosynthesis (Kruse *et al.*, 2018). Thus, ATM3 represents a hub linking Fe and N metabolism. Nevertheless, neither *atm3* nor *grxs17* is lethal in Arabidopsis (Balk and Pilon, 2011; Iñigo *et al.*, 2016); thus, the operation of the CIA system for the cytoplasmic biosynthesis of Fe–S clusters is not an exclusive source of the cofactors in the cytoplasm, and GRXS17 primarily operates in the maturation of cytoplasmic Fe–S proteins (Martins *et al.*, 2020). Recent results (Cheng *et al.*, 2020) on the expression of the Fe-responsive pathway elements showed, however, that GRXS17 can be also involved in the signalling of both the redox and Fe status. Since Fe liberation has a pivotal role in generating oxidative stress, the management of Fe–S clusters by GRXs is crucial in protecting them from Fe liberation. In consequence, redox signals can also report on improper Fe–S cluster metabolism.

Although the connections between the cytoplasmic and organellar Fe–S cluster metabolisms have not been revealed completely in plants, the comparison of results obtained in *Δatm3*, *Δgrxs17*, and *NEET-H89C* lines indicate that chloroplasts are important sources of Fe–S clusters for cytoplasmic proteins. Since NEET proteins are involved in the donation of 2Fe–2S clusters to cytoplasmic acceptors, they are suggested to have a role in the transfer of Fe–S clusters to cytoplasmic apoproteins (Karmi *et al.*, 2018; Zandalinas *et al.*, 2020; Nechushtai *et al.*, 2020). In Arabidopsis, NEET is double-localized to mitochondria and chloroplasts (Fig. 2; Nechushtai *et al.*, 2012; Su *et al.*, 2013; Khan *et al.*, 2018). Loss of NEET function is detrimental and results in enhanced Fe overaccumulation and reactive oxygen species (ROS) production, developmental retardation, enhanced senescence, increased sensitivity to low Fe nutrition, and decreased sensitivity to high Fe nutrition (Nechushtai *et al.*, 2012; Lu, 2018; Zandalinas *et al.*, 2020). The mechanism involved in the sensing of Fe–S clusters is also under debate in photosynthetically active plant cells.

Interaction between Fe(II) or Fe(III), nitric oxide (NO), and small-molecular-weight thiols such as GSH yields mononitrosyl Fe complexes (MNICs) and dinitrosyl Fe complexes (DNICs) (Lewandowska *et al.*, 2011; Li and Li, 2016). DNICs are formed during the attack by NO on Fe–S clusters that induces the release of Fe from the clusters (Landry *et al.*, 2011; Berndt and Lillig, 2017; Tewari *et al.*, 2021). GSH complexing of DNICs results in the increased stability of these species (Vanin, 2009). The connection between plant cells through plasmodesmata enables the symplastic transport of DNICs, so they could be also involved in medium/long-distance Fe delivery and signalling. Nevertheless, the effects of NO on Fe homeostasis seems to be tissue and developmental stage specific. In these signalling events, the connections of the nitrosative and Fe-sensing-induced signalling remain unresolved at multiple points.

## Compartmentalization of iron in leaves

The labile Fe pool is the source of Fe for organellar Fe uptake and cytoplasmic biosynthesis of cofactors (Fig. 2). Roschzttardtz *et al.* (2013) reported that, under normal Fe supply, Fe in the leaves is primarily localized to the chloroplasts of the spongy mesophyll cells and in the vasculature. Under supraoptimal Fe supply, the accumulation of Fe in these locations is more pronounced. Immune localization revealed that under normal Fe supply, ferritin is mainly located in the xylem-associated cells but not in mesophyll cells (Roschzttardtz *et al.*, 2013); thus, the presence of Fe in the chloroplasts of the spongy parenchyma cells corresponds to Fe that has already been incorporated into the photosynthetic machinery. The high-efficacy Fe uptake of chloroplasts relies on the reduction-based mechanism (Solti *et al.*, 2014; Sági-Kazár *et al.*, 2021). This machinery is thought to include the chloroplast inner envelope membrane proteins PIC1/Translocon of Inner Chloroplast envelope 21, Nickel-Cobalt Transporter, and Ferric Reductase Oxidase (FRO) 7 (Duy et al., 2007, 2011; Jeong *et al.*, 2008; Fig. 2). Although Asc is relatively abundant in chloroplasts and can reduce Fe(III) directly, it does not facilitate the reduction-based acquisition of Fe (Solti *et al.*, 2012). Since Fe(II) was not detected by ^57^Fe Mössbauer spectroscopy either in the intermembrane space or in the chloroplast stroma during Fe uptake (Solti *et al.*, 2012), the direct loading of Fe into transporters following the reduction step suggests the close collaboration of the Fe(III) reduction and Fe uptake machinery. This model also explains why externally applied Asc does not facilitate Fe uptake. Ferroportin (FPN) 3 (synonymous to IREG3 and MAR1; Conte *et al.* 2009) was identified as a double-localized transporter in both mitochondria and plastids (Fig. 2) (Kim *et al.*, 2021). FPN3 was suggested to be involved in NA uptake into the plastids (Conte and Lloyd, 2010; Duy *et al.*, 2011). Although in the shoot FPN3 expression is independent of the Fe status, it was suggested to have a role in Fe mobilization from organelles (Kim *et al.*, 2021). This role of NA in the chloroplasts is also supported by the Fe precipitation in the chloroplast stroma in NA-defective *chloronerva* tomatoes, as discussed above. Divol *et al.* (2013) showed that the Fe-NA transporters YSL4/YSL6 are involved in the Fe transport of plastids in germinating seeds, while Fe accumulates in the chloroplasts of *ysl4ysl6* double mutants. Nevertheless, *YSL4* expression remained low in *Brassica napus* leaves (Müller *et al.*, 2019) and no data are available on the functional characterization of YSL4/YSL6; it has been suggested they may have a role in Fe release from plastids (Fig. 2). Similarly, Mitoferrin-Like 1 (MFL1) (Fig. 2) remains a less well characterized component of the Fe homeostasis of chloroplasts (for review, see Vigani *et al.*, 2019). The plastidial, soluble, ATP-binding ABC-transporter subunits ABCI10 and ABCI11/NAP14 are also strongly associated with the Fe homeostasis of chloroplasts (Fig. 2) (Voith von Voithenberg *et al.*, 2019). The inner-envelope-associated ABCI10 also interacts with the membrane-intrinsic ABCI12, and this complex was suggested to be a part of an energy-coupled transporter unit (Voith von Voithenberg *et al.*, 2019). Nevertheless, the characterization of the mechanism of action of this system, including the direction of the transport, is still incomplete. Although the release of Fe from chloroplasts is evident during senescence (Barton, 1970; Pottier *et al.*, 2019; Pham *et al.*, 2020; Sági-Kazár *et al.*, 2021), it is not clear whether the export of Fe or Fe cofactors occurs. Moreover, the transport system that would be responsible for Fe release is also under debate. Nechushtai *et al.* (2020) suggests that the outer-envelope-anchored plastidial NEET may be involved in the delivery of 2Fe–2S clusters to cytoplasmic proteins. Nevertheless, the 2Fe–2S cluster transfer activity is restricted to cytoplasmic proteins, whereas no transmembrane transport activity has been confirmed so far, and thus its contribution to the export of Fe–S clusters out of the chloroplasts remains questionable.

**Fig. 3.**
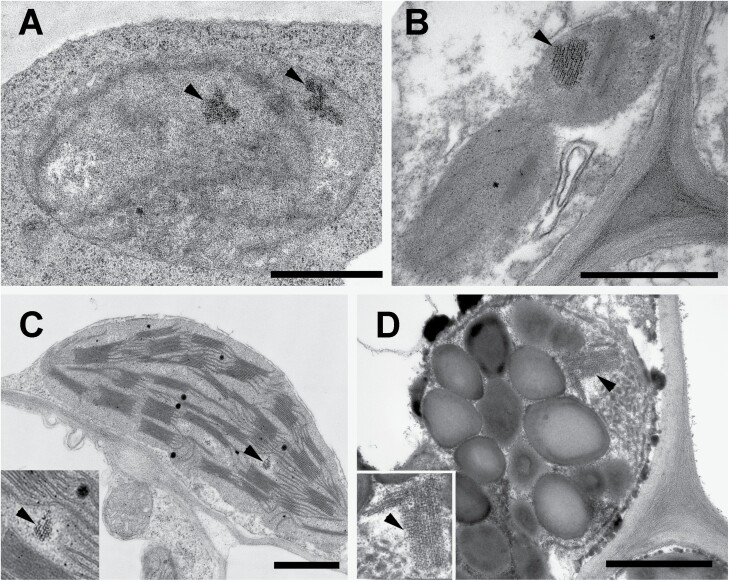
Localization of ferritin om various plastid types, cells and tissues of dicots. (A) Ferritins in an etio-chloroplast of a mesophyll cell in the outer leaf primordium of the fully closed bud of common ash (*Fraxinus excelsior*) (for further details see Solymosi *et al.*, 2012). (B) Ferritin from a plastid located in the phloem parenchyma of the leaves of 2-week-old dark-forced rosemary (*Rosmarinus officinalis*) shoot (for details see Böszörményi *et al.*, 2020). (C) Chloroplast with ferritin from the leaves of 2-week-old photosynthetically active light-grown pea (*Pisum sativum*). (D) Senescing chloroplast from the mesophyll (spongy parenchyma) cells of senescent *Parthenocissus tricuspidata* leaf. The arrowheads point to ferritin, and the insets show magnified views of the ferritin region of the plastids. Electron microscopic sample preparation and analysis were performed as described in Böszörményi *et al.* (2020). Scale bars=1 μm.

In aerial tissues, 80–90% of Fe is found in chloroplasts (Terry and Abadía, 1986), especially in photosystem (PS) I, PSII, and cytochrome *b*_6_*f* complexes of the photosynthetic electron transport chain, but also in redox enzymes of the nitrite and sulfate assimilation pathways (for review, see Hantzis *et al.*, 2018). Non-heme mononuclear Fe is an essential cofactor of the photosystem PSII core complex bound on His residues (Kato *et al.*, 2021). Therefore, Fe accumulation in chloroplasts has a primary importance in the Fe nutrition of mesophyll cells. According to chloroplast Fe uptake measurements, no Fe environments can be identified other than 4Fe–4S clusters, indicating that after Fe is taken up into the chloroplasts it is directly incorporated into Fe–S clusters (Solti *et al.*, 2012). Zhang *et al.* (2021) demonstrated that DJA5 and DJA6, mutation of which is lethal, bind Fe at conserved Cys residues and transfer it towards the SUF machinery. Under Fe deficiency, the SUF apparatus becomes suppressed due to down-regulation of *SUFA* and *SUFB* (Hantzis *et al.*, 2018; Sági-Kazár *et al.*, 2021), indicating that the SUF machinery stays under the regulation of intracellular Fe status. Moreover, chlorophyll accumulation is also tightly linked to Fe–S cluster biosynthesis (Hu *et al.*, 2017). Among the components of the photosynthetic machinery, cytochrome *b*_6_*f* and PSI are also highly affected by Fe deficiency (Hantzis *et al.*, 2018). Indeed, the amount of PSI supercomplexes depends strongly on Fe status (Andaluz *et al.*, 2006; Timperio *et al.*, 2007; Basa *et al.*, 2014). When the incorporation of Fe into the photosynthetic apparatus is restricted, such as under etiolation or during senescence, or when there is an excess of Fe, ferritins become the most significant Fe-containing components in chloroplasts (Fig. 3) (Roschzttardtz *et al.*, 2013; Hantzis *et al.*, 2018; Chen *et al.*, 2019). In Arabidopsis, FER1 and FER3 are plastidial proteins, FER4 is localized to plastids and mitochondria in vegetative tissues, whereas FER2 is found exclusively in seed plastids (Fig. 2) ([Bibr CIT0129][Bibr CIT0177]; Ravet *et al.*, 2009; Zhao, 2010; Theil, 2013). Since no ferritin-related Fe species were detected by ^57^Fe Mössbauer spectroscopy in chloroplasts originating from developed, non-senescent leaves of plants with optimal Fe nutrition (Solti *et al.*, 2012), the induction of ferritin-based Fe storage is negligible under these conditions. Instead, ferritins are involved in protection against oxidative damage by sequestering Fe in an inert form to avoid Fenton reactions (Briat and Lobréaux, 1997; Briat *et al.*, 1999; Ravet *et al.*, 2009). This protective function is important in the case of local or temporal excess of Fe (Izaguirre-Mayoral and Sinclair, 2009; Briat *et al.*, 2010; Roschzttardtz *et al.*, 2013). Ferritins have been observed by transmission electron microscopy in various cell types with photosynthetic apparatus that is not fully active, such as plastids in xylem- or phloem-associated parenchyma (Fig. 3) (Tarantino *et al.*, 2003; Böszörményi *et al.*, 2020; Roschzttardtz *et al.*, 2013). Ferritin also occurs in plastids of etiolated organs such as leaf primordia of cabbage (*Brassica oleracea*) (Solymosi *et al.*, 2004) or buds (Fig. 3) (Solymosi *et al.*, 2012). Temporal Fe excess in plastids, and thus ferritin accumulation, is observed during normal plant development, including leaf senescence (Fig. 3) (Tarantino *et al.*, 2003; Solymosi *et al.*, 2004), but also under various stress conditions (Seckback, 1982). During senescence, ferritins operate as temporary storage of Fe released from the breakdown of the photosynthetic apparatus before its remobilization (Fig. 3). Frataxin seems to have an overlapping role with ferritins in keeping cellular Fe levels under control (Ramirez *et al.*, 2011; Murgia and Vigani, 2015). Nevertheless, the relationship of ferritin- and frataxin-based Fe storage in cells is not clear in the leaf. Ferritin-based Fe storage in plastids, indeed, seems to be associated with situations involving a high risk of Fe liberation. Thus, ferritins are predominant in binding Fe in the organelles, especially in chloroplasts, whereas mobile Fe ligands such as NA are instead involved in the mediation of Fe loading to and release from ferritins.

In mitochondria, elements of the respiratory electron transport chain also require a significant amount of Fe in the form of cofactors (for review, see Vigani and Hanikenne, 2018). Similarly to plastids, the Fe transport activities of mitochondria also rely on the reduction-based strategy (Fig. 2). *In silico* analysis predicts two FRO proteins (FRO3 and FRO8) that target mitochondria in Arabidopsis (Mukherjee *et al.*, 2006; Connolly *et al.*, 2018). FRO3, which is also expressed in the vascular cylinder of roots (Mukherjee *et al.*, 2006), is abundant during leaf development, but the accumulation of FRO8 is restricted to senescence in shoots; thus, a functional split between mitochondrial FROs exists (Vigani *et al.*, 2019). In contrast to chloroplast FRO7, which is localized in the inner envelope of chloroplasts (Solti *et al.*, 2014), *in silico* analysis suggests that FRO3 may target the outer membrane of mitochondria and thus can utilize cytosolic NADH (Connolly *et al.*, 2018). Indeed, these preliminary data require validation. Altogether, functional analysis of mitochondrial FRO enzymes remains incomplete. Once Fe(III) is reduced, it should cross the mitochondrial membranes. A mitochondrial Fe transporter (MIT) has been characterized in rice (*Oryza sativa*) (Bashir *et al.*, 2011; Vigani *et al.*, 2016). Arabidopsis expresses two Mitoferrin (MIT) proteins, MIT1 and MIT2 (Fig. 2; Jain *et al.*, 2019). The *mit1* and *mit2* single mutants show no visible phenotype, whereas the *mit1mit2* double mutation is lethal (Jain *et al.*, 2019). It is important to note that a significant proportion of available data on mitochondrial Fe homeostasis has been obtained from root cells, and validation of these data is also required in photosynthetically active cells.

Mesophyll cells are characterized by the presence of a central vacuole, which is involved in the regulation of cellular Fe homeostasis (Gayomba *et al.*, 2015; Vigani *et al.*, 2019; Bashir *et al.*, 2021). Fe loading into the vacuole is primarily managed by Vacuolar Iron Transporter (VIT) proteins (Eroglu *et al.*, 2019). In rice, *OsVIT1* is expressed in developing leaves (Zhang *et al.*, 2012; Che *et al.*, 2021). Functionally redundant VIT-like (VTL) transporter genes *VTL1*, *VTL2*, and *VTL5* are expressed in vegetative tissues (Fig. 2) (Gollhofer *et al.*, 2014). Knowledge of the exact histological location of VIT and VTL proteins in leaves is still scarce, although VIT1 was shown to be active in the xylem parenchyma cells of Arabidopsis embryos (Kim *et al.*, 2006). Although clear evidence is missing on the vacuolar Fe compounds, it is likely that Fe(III) associates with carboxylates (Roschzttardtz *et al.*, 2009; Flis *et al.*, 2016; Vigani *et al.*, 2019). NRAMP3 and NRAMP4 are involved in the retrieval of Fe (Fig. 2) (Lanquar et al., 2005; 2010; Bastow *et al.*, 2018). Although vacuolar Fe exporters are suggested to be divalent metal carriers, the mechanism of Fe reduction for the transport is still unclear. So far, no FRO enzymes have been identified in the tonoplast membrane in Arabidopsis, although OsFRO1 was shown to act as a vacuolar ferric chelate reductase in rice leaves (Li *et al.*, 2019). Overexpression of OsFRO1 leads to increased Fe sensitivity, while Fe excess leads to the down-regulation of OsFRO1 (Li *et al.*, 2019). Asc-mediated Fe reduction can be also important in mobilizing vacuolar Fe. In Arabidopsis, the MATE family member DTX25 was shown to transport Asc across the tonoplast membrane, and its mutation caused sensitivity to Fe deficiency during germination (Fig. 2) (Hoang *et al.*, 2021). As a consequence, higher plants seem to transport reduced Fe across the tonoplast membrane. The means of Fe accumulation and reduction is believed to be taxon dependent, but the transport of reduced Fe is supported by evidence. Vacuolar Fe storage becomes important when ferritin-based storage in the organelles is limited, but it can be also involved in Fe reallocation processes associated with the degradation of cell compartments, discussed below.

## Information on the intracellular iron status in leaves

Fe uptake and allocation in the cells require the sensing of intracellular Fe status, inducing a reciprocal interaction between sensing and regulation. In contrast to root cells, which take up Fe and translocate it to the places where it is utilized, leaf cells—especially during leaf development—are the sink of Fe trafficking. Since the overaccumulation of Fe, especially in the presence of ROS, can lead to toxicity, controlling this accumulation is essential. Taking into account the complexity of cellular Fe homeostasis, and physiological and developmental responses to Fe, multiple Fe-sensing mechanisms should be involved in the fine-tuning of Fe homeostasis (Miller and Busch, 2021). Conserved mechanisms in protein-based Fe signalling in eukaryotic cells suggest that the mechanism of monitoring intracellular Fe status has a common nature.

Although the nature and composition of the labile Fe pool in the cytoplasm is still under debate, sensing the available Fe pool is of primary importance. In the sensing of intracellular Fe, O-bridged di-Fe clusters bound to hemerythrin-type proteins have a primary importance. Hemerythrin motif-containing RING and zinc-finger proteins (HRZs) in rice and BRUTUS (BTS) E3 ubiquitin ligases in Arabidopsis are negative regulators of Fe deficiency responses (Fig. 2) (Long *et al.*, 2010; Hindt *et al.*, 2017; Kobayashi, 2019; Riaz and Guerinot, 2021). The function of Arabidopsis BTS is redundant to that of its paralogs, BTS-Like (BTSL) 1 and BTSL2, but in leaves, only *BTS* is expressed (Rodríguez-Celma *et al.*, 2019). Based on the tissue-specific differences in the expression of hemerythrin-type sensors, slight alterations in the Fe signalling among root and shoot tissues can be expected. Fe binding to BTS directs IVc basic helix-loop-helix (bHLH) transcription factors, upstream regulators of Fe signalling, to degradation (Rodríguez-Celma *et al.*, 2019; Gao and Dubos, 2021). The expression of *HRZs/BTS* is strongly induced by Fe deficiency in shoot tissues (Rodríguez-Celma *et al.*, 2013; Kobayashi *et al.*, 2013; Hindt *et al.*, 2017). Although the Fe signalling cascade of bHLH transcription factors is well established in roots, the role of bHLH transcription factors in the regulation of Fe homeostasis in leaves remains controversial. The *in silico* analysis of Hantzis *et al.* (2018) indicated that major Fe homeostasis effector elements in leaves such as *SUFB* and *Ferredoxin 2* have no or a very limited probability of bHLH binding to their promoter regions. It is also an open question whether organellar Fe status regulates the expression of nuclear genes directly.

In the Fe homeostasis of leaf cells, signals derived from the Fe and the associated metabolic status of organelles could have primary importance. Both chloroplasts and mitochondria synthesize 3ʹ-phosphoadenisine-5ʹ-phosphate (PAP), which is involved in retrograde signalling (Estavillo *et al.*, 2011). Mutation of the PAP system induces constant activation of the root Fe acquisition system together with higher shoot Fe accumulation (Balparda *et al.*, 2020). Since organelles are the primary sites of Fe incorporation and cofactor biosynthesis, the saturation of which is required for a balanced metabolism, the PAP system is a supposed member of the retrograde signalling system that also delivers information on the Fe status. Nevertheless, operation of the PAP system needs further investigations in photosynthetically active cells. The elimination of formate also seems to be linked to the signalling of organellar Fe status. Formate is produced in the methionine cycle and is involved in NA biosynthesis. Its detoxification relies on Formate Dehydrogenase (FDH), a double-localized protein in the mitochondria and chloroplasts. Together with NA biosynthesis, FDH is also induced by Fe deficiency (Suzuki *et al.*, 1998); the FDH promoter is sensitive to Fe status, and changes in *FDH* expression lead to altered Fe accumulation in aerial tissues (Vigani *et al.*, 2017, Di Silvestre *et al.*, 2021). In consequence, formate can act as an indirect signal of intracellular Fe status.

In addition to PAP and formate, NO metabolism is also linked to Fe status signalling in leaves. Upon Fe excess, rapid NO accumulation occurs in the chloroplasts (Arnaud *et al.*, 2006). Excess Fe was shown to decrease the expression of *FRO7* (Sági-Kazár *et al.*, 2021). Forming *S*-nitrosoglutathione adduct with GSH, a nitrosative signal triggers the Fe acquisition system in roots (Kailasam *et al.*, 2018). Under optimal Fe nutrition, overexpression of *S*-nitrosoglutathione reductase (GSNOR), which eliminates *S*-nitrosoglutathione and thus the nitrosative signal, was associated with a decrease in the expression of *FER1*, *FER2*, *PIC*, *FRO7*, and *VIT* in mature leaves of tomato (Wen *et al.*, 2019). In contrast, under Fe deficiency, overexpression of *GSNOR* significantly up-regulated the expression of *FRO7* (Wen *et al.*, 2019). Since in developed leaves the expression of *FRO7* is suppressed under conditions of both Fe deficiency and Fe excess (Sági-Kazár *et al.*, 2021), the nitrosative signal seems to be a negative regulator of the expression of plastidial Fe acquisition elements. Although NO biosynthesis in chloroplasts still remains unresolved, NO-induced signals directly impact the Fe homeostasis. It seems very likely that the regulatory role of plastidial Fe content on Fe acquisition by chloroplasts (Solti *et al.*, 2012) could also rely on a nitrosative signal. In accordance, the activity of FRO7, a key component in the reduction-based Fe acquisition mechanism of chloroplasts, is suppressed by a slight excess of Fe (Sági-Kazár *et al.*, 2021). Nevertheless, data on the effect of the nitrosative signal on cellular Fe homeostasis still remains controversial, that is, the nitrosative signal seems to be a positive regulator of organellar Fe allocation and Fe storage under optimal Fe nutrition, whereas under Fe deficiency or excess, it is suggested to limit at least Fe allocation to the chloroplasts. In plastids, Fe–S clusters may also be connected with signalling processes. Fe–S cluster biosynthesis is supposedly involved in the feedback suppression of plastidial Fe uptake. The NAP1–NAP7–NAP6 complex, a component of the SUF system, is considered to provide a signal of the Fe status of plastids (for review, see Briat *et al.*, 2007).

It has also been long debated whether aconitase (ACO) or any other Fe–S cluster redox enzyme fulfils a sensor role in plants. For comparison, the cytoplasmic 4Fe–4S redox enzyme ACO monitors the cytoplasmic availability of Fe–S clusters and regulates the expression of *FER* and *Transferrin* genes in human cells (Hernández-Gallardo and Missirlis, 2020; Garza *et al.*, 2020; Senoura *et al.*, 2020). Based on the *aco1-3* mutant, Arnaud *et al.* (2007) indicated that the regulation of Fe homeostasis by ACO is unlikely in Arabidopsis. In contrast, Senoura *et al.* (2020) reported that rice ACO1 binds RNA and performs upstream regulation of the Fe acquisition transcription factors. Therefore, the Fe signalling mechanisms seem to be taxon specific, and further investigations are required to elucidate the impact of plant ACO on Fe homeostasis across angiosperms.

Intracellular Fe status is in a dynamic and reciprocal interaction with epigenetic regulation, which is involved in the establishment of the complexity of responses to altered Fe nutrition (Shafiq *et al.*, 2020). DNA methylation regulates transcriptional activity (Harris *et al.*, 2018). Sun *et al.* (2021) showed that DNA methylation is important in the induction of Fe-deficiency responses in the roots. Asymmetric DNA methylation of the plant genome is guided by non-coding RNAs (Zhang *et al.*, 2018), the expression pattern of which alters under Fe deprivation (Wang *et al.*, 2021). Although in animal models, the Fe sensitivity of DNA demethylation is also reported (Wang *et al.*, 2018; Camarena *et al.*, 2021), such a mechanism has not yet been reported in plants. As well as DNA methylation, histone modifications, namely acetylation and methylation, are involved in the regulation of Fe homeostasis (Fig. 2). Histone methylation is a repressive mark placed by the Polycomb Repressive Complex 2 (PRC2). The histone methylation pattern is altered under Fe excess in roots; the repressive marks arginine dimethylation H4Arg3me2 and lysine trimethylation H3Lys27me3 induced the suppression of the Fe acquisition system (Séré and Martin, 2020). In the shoot, under conditions of Fe excess, PRC2 targets *YSL1* and *IRON MAN1*, both of which are involved in long-distance Fe signalling (Kumar *et al.*, 2017; Grillet *et al.*, 2018). Although the information on the effect of epigenetic changes on Fe homeostasis in leaf cells is limited, suppressing the function of PRC2 affects the expression of both *FRO7* (positively) and *BTSL1* (negatively under Fe deficiency; Park *et al.*, 2020). Trimethylation of H3Lys4 is also essential in the regulation of Fe-deficiency responses. *FER1*, *FER3*, and *FER14* have promoter H3Lys4me3 signs under Fe excess (Tissot *et al.*, 2019), although the placing of this signal is regulated by as yet unknown processes. Since the ferritin-based Fe storage is of primary importance under multiple physiological conditions, epigenetic regulation of the expression of *FER* genes represents an important, superior regulation of Fe homeostasis as well as the amount of Fe that can accumulate in the leaf cells. In contrast to methylation, histone acetylation makes the DNA chains better available. The histone acetyltransferase enzyme General Control Non-repressed 5 was shown to be involved in H3Lys9 and H3Lys14 acetylation at the *FRD3* site (Xing *et al.*, 2015); thus, histone acetylation affects root-to-shoot Fe translocation. Although different histone modification mechanisms are known to affect both signalling and effector elements of root Fe homeostasis, information is limited in relation to leaves. Nevertheless, available data and evidence from animal/yeast cells suggest that cellular Fe nutrition generally provokes the alteration of epigenetic markers.

## Alteration of foliar iron homeostasis during leaf senescence

The generative processes of monocarpic plants lead to alterations in Fe allocation (Martínez-Ballesta *et al.*, 2020). During leaf senescence, a significant amount of leaf Fe content can be redistributed towards the shoot apex (Zhang *et al.*, 1995), although the extent to which this occurs is rather taxon specific. The re-translocation of Fe on a source–sink basis from leaves towards developing tissues should operate on a symplastic/phloem translocation pathway. In consequence, the biosynthesis of NA is enhanced in leaves during senescence (Shi *et al.*, 2012). Since the degradation of the photosynthetic apparatus and chloroplasts is an important process during leaf senescence (Domínguez and Cejudo, 2021), a controlled dismounting of Fe cofactors from the photosynthetic machinery is required. In parallel with this process, Fe-storing ferritin appears in plastids (Barton, 1970; Fig. 3). Autophagy is an important process in the remodelling of tissues and cell contents. Recent data suggest that vacuolar autophagy of chloroplasts (chlorophagy) is essential in Fe relocation (Pottier *et al.*, 2019). In the autophagy *atg5-1* mutant, Fe translocation from vegetative to generative tissues is reduced (Mari *et al.*, 2020). Based on a comparison with other autophagy-defective mutants, a two-step re-translocation model was suggested where Fe first accumulates in vegetative organs and subsequently remobilizes to seeds (Pottier *et al.*, 2019). Nevertheless, autophagy processes should be accompanied by Fe relocation within the cells, with Fe liberated from the photosynthetic apparatus being accumulated at least temporarily in lytic vacuoles. In consequence, the degradation of the photosynthetic apparatus serves as a source of Fe for re-translocation towards sink tissues from senescing leaves; however, a better understanding of intracellular Fe relocation processes is still required.

## Concluding remarks

In mesophyll cells, the transport and homeostasis of Fe is dominated by the proper incorporation of Fe into the photosynthetic electron transport chain. Although in the past 15 years multiple aspects of plastidial Fe homeostasis have been revealed, there is less in the literature concerning the mitochondria of photosynthetically active cells, and thus information on mitochondrial Fe homeostasis is mainly based on root cells. The loading of Fe into redox-active sites has a danger of uncontrolled Fenton reactions; thus, the proper complexing of Fe is essential. Fe–S clusters are among the most important Fe-containing cofactors. Indeed, further investigations are required on their transport across organellar membranes in mesophyll cells to reveal whether, and to what extent, the organellar Fe–S clusters contribute to the formation of cytoplasmic holoproteins. In the control of cellular and organellar Fe homeostasis, Fe-sensing mechanisms have a pivotal role. Although hemerythrin-domain Fe sensing exists in foliar cells, the understanding of intracellular, and especially organellar, Fe status is far from complete. The amount of data on the connection between epigenetic markers and the Fe status is increasing. However, the mechanisms that lead to the alteration of the epigenetic signs have not been revealed yet. The processing of Fe cofactors in photosynthetically active cells share similarities between mitochondria and plastids, but the coordination of mitochondrial and plastidial Fe homeostasis has not been resolved yet. Finally, Fe homeostasis in leaves undergoes an alteration during senescence, when the leaf status changes from a sink to a source of Fe for developing and generative tissues. Nevertheless, little is known about the intracellular processing of Fe during senescence processes, including the liberation of Fe from the cofactors and the alterations of intracellular Fe transport. Since Fe remobilization from leaves affects both plant growth and crop yield and also the holistic effectiveness of foliar Fe treatment, revealing these intracellular processes will have a high impact in the future.

**Box 1. Iron transport and homeostasis of photosynthetically active cells in leaves support the need for iron in chloroplasts.**

Higher plants primarily take up Fe by their roots, but due to the high demand for Fe to build up and operate the photosynthetic apparatus in the chloroplasts of photosynthetically active tissues, the largest proportion of Fe is translocated to the leaves. The high demand for Fe in the photosynthetic apparatus is supported by the Fe-carboxylates translocated in the xylem sap and taken up by leaf cells through the reduction-based Fe transport strategy. Once Fe is taken up by the cells, it becomes part of the labile Fe pool of the cytoplasm. Characterization of this pool has multiple technical difficulties since the concentration of Fe remains below the threshold of Fe speciation techniques and isolation of Fe with intact micro surrounding has not been resolved either. Although Fe has a primary importance in photosynthesis, mitochondrial function also requires a significant amount of Fe. In comparison to roots, where Fe management is dominated by the acquisition of Fe and its translocation towards sink tissues, Fe homeostasis of leaves is for the most part dependent on the incorporation of Fe into cofactors of redox enzymes. Nevertheless, the direction of the intracellular Fe allocation in leaves is also dependent on the developmental programme. During leaf development, the biosynthesis of the photosynthetic apparatus dominates. Information on Fe management in leaf primordia before the intensive development of chloroplasts and their photosynthetic apparatus is highly limited. Fully developed mature leaves support the carbon, nitrogen, and sulfur autotrophy of the plant. Sooner or later, leaves turn to senescence and become a source of organic and inorganic material. During senescence, Fe is liberated from the photosynthetic apparatus, leading to an intracellular reallocation of Fe and a remobilization towards new sink tissues. Since the vegetative parts of plants are also important in human nutrition, understanding Fe homeostasis and its regulation in leaves could also lead to improved plant breeding and pave the way for high-precision agriculture techniques. Moreover, understanding the mechanisms of Fe remobilization from senescing leaves could also lead to enhanced efficiency in foliar Fe fertilization.

## Abbreviations

Asc: Ascorbate
Cit: citrate
DNIC: dinitrosyl Fe complexes
GSH: glutathione
Mal: malate
MNIC: mononitrosyl Fe complexes
NA: nicotianamine
ROS: reactive oxygen species

## References

[CIT0001] Andaluz S , López-Millán AF, De las Rivas J, Aro EM, Abadía J, Abadía A. 2006. Proteomic profiles of thylakoid membranes and changes in response to iron deficiency. Photosynthesis Research 89, 141–155. 16969715 10.1007/s11120-006-9092-6

[CIT0002] Anderegg G , Ripperger H. 1989. Correlation between metal complex formation and biological activity of nicotianamine analogues. Journal of the Chemical Society, Chemical Communications 10, 647–650.

[CIT0003] Aravind P , Prasad MN. 2005. Cadmium-induced toxicity reversal by zinc in *Ceratophyllum demersum* L. (a free floating aquatic macrophyte) together with exogenous supplements of amino- and organic acids. Chemosphere 61, 1720–1733. 15907970 10.1016/j.chemosphere.2005.03.088

[CIT0004] Armas AM , Balparda M, Terenzi A, Busi MV, Pagani MA, Gomez-Casati DF. 2019. Ferrochelatase activity of plant frataxin. Biochimie 156, 118–122. 30342111 10.1016/j.biochi.2018.10.009

[CIT0005] Armas AM , Balparda M, Terenzi A, Busi MV, Pagani MA, Gomez-Casati DF. 2020. Iron-sulfur cluster complex assembly in the mitochondria of *Arabidopsis thaliana*. Plants 9, 1171. 10.3390/plants9091171 PMC7570111 32917022

[CIT0006] Arnaud N , Murgia I, Boucherez J, Briat JF, Cellier F, Gaymard F. 2006. An iron-induced nitric oxide burst precedes ubiquitin-dependent protein degradation for *Arabidopsis AtFer1* ferritin gene expression. Journal of Biological Chemistry 281, 23579–23588. 16782706 10.1074/jbc.M602135200

[CIT0007] Arnaud N , Ravet K, Borlotti A, Touraine B, Boucherez J, Fizames C, Briat JF, Cellier F, Gaymard F. 2007. The iron-responsive element (IRE)/iron-regulatory protein 1 (IRP1)–cytosolic aconitase iron-regulatory switch does not operate in plants. Biochemical Journal 405, 523–531. 17437406 10.1042/BJ20061874 PMC2267314

[CIT0008] Askenasy I , Stroupe ME. 2020. The siroheme-[4Fe–4S] coupled center. In: Torres MS, Kroneck P, eds. Transition metals and sulfur - a strong relationship for life. Berlin, Boston: De Gruyter, 343–380.

[CIT0009] Balk J , Pilon M. 2011. Ancient and essential: the assembly of iron–sulfur clusters in plants. Trends in Plant Science 16, 218–226. 21257336 10.1016/j.tplants.2010.12.006

[CIT0010] Balk J , Schaedler TA. 2014. Iron cofactor assembly in plants. Annual Review of Plant Biology 65, 125–153. 10.1146/annurev-arplant-050213-035759 24498975

[CIT0011] Balparda M , Armas AM, Estavillo GM, Roschzttardtz H, Pagani MA, Gomez-Casati DF. 2020. The PAP/SAL1 retrograde signaling pathway is involved in iron homeostasis. Plant Molecular Biology 102, 323–337. 31900819 10.1007/s11103-019-00950-7

[CIT0012] Barton R. 1970. The production and behaviour of phytoferritin particles during senescence of *Phaseolus* leaves. Planta 94, 73–77. 24496818 10.1007/BF00386610

[CIT0013] Basa B , Lattanzio G, Solti Á, Tóth B, Abadía J, Fodor F, Sárvári É. 2014. Changes induced by cadmium stress and iron deficiency in the composition and organization of thylakoid complexes in sugar beet (*Beta vulgaris* L.). Environmental and Experimental Botany 101, 1–11.

[CIT0014] Bashir K , Ahmad Z, Kobayashi T, Seki M, Nishizawa NK. 2021. Roles of subcellular metal homeostasis in crop improvement. Journal of Experimental Botany 72, 2083–2098. 33502492 10.1093/jxb/erab018

[CIT0015] Bashir K , Ishimaru Y, Shimo H, Nagasaka S, Fujimoto M, Takanashi H, Tsutsumi N, An G, Nakanishi H, Nishizawa NK. 2011. The rice mitochondrial iron transporter is essential for plant growth. Nature Communications 2, 322. 10.1038/ncomms1326 PMC3113228 21610725

[CIT0016] Bastow EL , Garcia de la Torre VS, Maclean AE, Green RT, Merlot S, Thomine S, Balk J. 2018. Vacuolar iron stores gated by NRAMP3 and NRAMP4 are the primary source of iron in germinating seeds. Plant Physiology 177, 1267–1276. 29784767 10.1104/pp.18.00478 PMC6052989

[CIT0017] Bauer P , Thiel T, Klatte M, Bereczky Z, Brumbarova T, Hell R, Grosse I. 2004. Analysis of sequence, map position, and gene expression reveals conserved essential genes for iron uptake in Arabidopsis and tomato. Plant Physiology 136, 4169–4183. 15531708 10.1104/pp.104.047233 PMC535847

[CIT0018] Becker R , Fritz E, Manteuffel R. 1995. Subcellular localization and characterization of excessive iron in the nicotianamine-less tomato mutant *chloronerva*. Plant Physiology 108, 269–275. 12228472 10.1104/pp.108.1.269 PMC157331

[CIT0019] Berndt C , Lillig CH. 2017. Glutathione, glutaredoxins, and iron. Antioxidants and Redox Signaling 27, 1235–1251. 28537421 10.1089/ars.2017.7132

[CIT0020] Bhattacharyya A , Schmidt MP, Stavitski E, Azimzadeh B, Martínez CE. 2019. Ligands representing important functional groups of natural organic matter facilitate Fe redox transformations and resulting binding environments. Geochimica et Cosmochimica Acta 251, 157–175.

[CIT0021] Bhattacharyya A , Stavitski E, Dvorak J, Martínez CE. 2013. Redox interactions between Fe and cysteine: spectroscopic studies and multiplet calculations. Geochimica et Cosmochimica Acta 122, 89–100.

[CIT0022] Böszörményi A , Dobi A, Skribanek A, Pávai M, Solymosi K. 2020. The effect of light on plastid differentiation, chlorophyll biosynthesis, and essential oil composition in rosemary (*Rosmarinus officinalis*) leaves and cotyledons. Frontiers in Plant Science 11, 196. 32194595 10.3389/fpls.2020.00196 PMC7063033

[CIT0023] Briat JF , Curie C, Gaymard F. 2007. Iron utilization and metabolism in plants. Current Opinion in Plant Biology, 10, 276–282. 17434791 10.1016/j.pbi.2007.04.003

[CIT0024] Briat JF , Lobréaux S. 1997. Iron transport and storage in plants. Trends in Plant Science 2, 187–193.

[CIT0025] Briat JF , Lobreaux S, Grignon N, Vansuyt G. 1999. Regulation of plant ferritin synthesis: how and why. Cellular and Molecular Life Sciences 56, 155–166. 11213255 10.1007/s000180050014 PMC11146809

[CIT0026] Briat JF , Ravet K, Arnaud N, Duc C, Boucherez J, Touraine B, Cellier F, Gaymard F. 2010. New insights into ferritin synthesis and function highlight a link between iron homeostasis and oxidative stress in plants. Annals of Botany 105, 811–822. 19482877 10.1093/aob/mcp128 PMC2859905

[CIT0027] Brüggemann W , Maas-Kantel K, Moog PR. 1993. Iron uptake by leaf mesophyll cells: the role of the plasma membrane-bound ferric-chelate reductase. Planta 190, 151–155.

[CIT0028] Brzezowski P , Richter AS, Grimm B. 2015. Regulation and function of tetrapyrrole biosynthesis in plants and algae. Biochimica et Biophysica Acta - Bioenergetics 1847, 968–985.

[CIT0029] Camarena V , Huff TC, Wang G. 2021. Epigenomic regulation by labile iron. Free Radical Biology and Medicine 170, 44–49. 33493555 10.1016/j.freeradbiomed.2021.01.026 PMC8217092

[CIT0030] Che J , Yamaji N, Ma JF. 2021. Role of a vacuolar iron transporter OsVIT2 in the distribution of iron to rice grains. New Phytologist 230, 1049–1062. 33474769 10.1111/nph.17219

[CIT0031] Chen J , Browne WR. 2018. Photochemistry of iron complexes. Coordination Chemistry Reviews 374, 15–35.

[CIT0032] Chen Q , Shinozaki D, Luo J, et al. 2019. Autophagy and nutrients management in plants. Cells 8, 1426. 10.3390/cells8111426 PMC6912637 31726766

[CIT0033] Cheng N , Yu H, Rao X, Park S, Connolly EL, Hirschi KD, Nakata PA. 2020. Alteration of iron responsive gene expression in Arabidopsis glutaredoxin *S17* loss of function plants with or without iron stress. Plant Signaling & Behavior 15, 1758455. 32351167 10.1080/15592324.2020.1758455 PMC8570760

[CIT0034] Clemens S , Deinlein U, Ahmadi H, Höreth S, Uraguchi S. 2013. Nicotianamine is a major player in plant Zn homeostasis. Biometals 26, 623–632. 23775667 10.1007/s10534-013-9643-1

[CIT0035] Cointry V , Vert G. 2019. The bifunctional transporter-receptor IRT 1 at the heart of metal sensing and signalling. New Phytologist 223, 1173–1178. 30929276 10.1111/nph.15826

[CIT0036] Connolly EL , Jain A, Juengst B. 2018. Integrating cellular iron compartmentalization mechanisms. In: Schmidt W, ed. 19th International Symposium on Iron Nutrition and Interactions in Plants. Taipei, Taiwan, July 9–13, 2018. Proceedings. Taipei: Academia Sinica, 41.

[CIT0037] Conte SS , Lloyd AM. 2010. The MAR1 transporter is an opportunistic entry point for antibiotics. Plant Signaling and Behavior 5, 49–52. 20592808 10.4161/psb.5.1.10142 PMC2835957

[CIT0038] Conte S , Stevenson D, Furner I, Lloyd A. 2009. Multiple antibiotic resistance in Arabidopsis is conferred by mutations in a chloroplast-localized transport protein. Plant Physiology 151, 559–573. 19675150 10.1104/pp.109.143487 PMC2754617

[CIT0039] Couturier J , Przybyla-Toscano J, Roret T, Didierjean C, Rouhier N. 2015. The roles of glutaredoxins ligating Fe–S clusters: sensing, transfer or repair functions? Biochimica et Biophysica Acta - Molecular Cell Research 1853, 1513–1527. 10.1016/j.bbamcr.2014.09.018 25264274

[CIT0040] Cui M , Gu M, Lu Y, Zhang Y, Chen C, Ling H-Q, Wu H. 2020. Glutamate synthase 1 is involved in iron-deficiency response and long-distance transportation in *Arabidopsis*. Journal of Integrative Plant Biology 62, 1925–1941. 32584503 10.1111/jipb.12985

[CIT0041] Curie C , Briat JF. 2003. Iron transport and signaling in plants. Annual Review of Plant Biology 54, 183–206. 10.1146/annurev.arplant.54.031902.135018 14509968

[CIT0042] Curie C , Cassin G, Couch D, et al. 2009. Metal movement within the plant: contribution of nicotianamine and yellow stripe 1-like transporters. Annals of Botany 103, 1–11. 18977764 10.1093/aob/mcn207 PMC2707284

[CIT0043] Di Silvestre D , Vigani G, Mauri P, Hammadi S, Morandini P, Murgia I. 2021. Network topological analysis for the identification of novel hubs in plant nutrition. Frontiers in Plant Science 12, 51. 10.3389/fpls.2021.629013 PMC7928335 33679842

[CIT0044] Divol F , Couch D, Conéjéro G, Roschzttardtz H, Mari S, Curie C. 2013. The *Arabidopsis* YELLOW STRIPE LIKE4 and 6 transporters control iron release from the chloroplast. The Plant Cell 25, 1040–1055. 23512854 10.1105/tpc.112.107672 PMC3634676

[CIT0045] Domínguez F , Cejudo FJ. 2021. Chloroplast dismantling in leaf senescence. Journal of Experimental Botany 72, 5905–5918. 33959761 10.1093/jxb/erab200 PMC8760853

[CIT0046] Durrett TP , Gassmann W, Rogers EE. 2007. The FRD3-mediated efflux of citrate into the root vasculature is necessary for efficient iron translocation. Plant Physiology 144, 197–205. 17351051 10.1104/pp.107.097162 PMC1913786

[CIT0047] Duy D , Stübe R, Wanner G, Philippar K. 2011. The chloroplast permease PIC1 regulates plant growth and development by directing homeostasis and transport of iron. Plant Physiology 155, 1709–1722. 21343424 10.1104/pp.110.170233 PMC3091129

[CIT0048] Duy D , Wanner G, Meda AR, von Wirén N, Soll J, Philippar K. 2007. PIC1, an ancient permease in *Arabidopsis* chloroplasts, mediates iron transport. The Plant Cell 19, 986–1006. 17337631 10.1105/tpc.106.047407 PMC1867359

[CIT0049] Eroglu S , Karaca N, Vogel-Mikus K, Kavčič A, Filiz E, Tanyolac B. 2019. The conservation of VIT1-dependent iron distribution in seeds. Frontiers in Plant Science 10, 907. 31354774 10.3389/fpls.2019.00907 PMC6640190

[CIT0050] Estavillo GM , Crisp PA, Pornsiriwong W, et al. 2011. Evidence for a SAL1-PAP chloroplast retrograde pathway that functions in drought and high light signaling in *Arabidopsis*. The Plant Cell 23, 3992–4012. 22128124 10.1105/tpc.111.091033 PMC3246320

[CIT0051] Flis P , Ouerdane L, Grillet L, Curie C, Mari S, Lobinski R. 2016. Inventory of metal complexes circulating in plant fluids: a reliable method based on HPLC coupled with dual elemental and high-resolution molecular mass spectrometric detection. New Phytologist 211, 1129–1141. 27111838 10.1111/nph.13964

[CIT0052] Gao F , Dubos C. 2021. Transcriptional integration of plant responses to iron availability. Journal of Experimental Botany 72, 2056–2070. 33246334 10.1093/jxb/eraa556

[CIT0053] Garza KR , Clarke SL, Ho YH, Bruss MD, Vasanthakumar A, Anderson SA, Eisenstein R S. 2020. Differential translational control of 5ʹ IRE-containing mRNA in response to dietary iron deficiency and acute iron overload. Metallomics 12, 2186–2198. 33325950 10.1039/d0mt00192a PMC8057200

[CIT0054] Gayomba SR , Zhai Z, Jung HI, Vatamaniuk OK. 2015. Local and systemic signaling of iron status and its interactions with homeostasis of other essential elements. Frontiers in Plant Science 6, 716. 26442030 10.3389/fpls.2015.00716 PMC4568396

[CIT0055] Goldberg RN , Kishore N, Lennen RM. 2002. Thermodynamic quantities for the ionization reactions of buffers. Journal of Physical and Chemical Reference Data 31, 231–370.

[CIT0056] Gollhofer J , Timofeev R, Lan P, Schmidt W, Buckhout TJ. 2014. Vacuolar-iron-transporter1-like proteins mediate iron homeostasis in Arabidopsis. PLoS One 9, e110468. 25360591 10.1371/journal.pone.0110468 PMC4215979

[CIT0057] Gomez-Casati DF , Busi MV, Pagani MA. 2018. Plant frataxin in metal metabolism. Frontiers in Plant Science 9, 1706. 30519254 10.3389/fpls.2018.01706 PMC6258813

[CIT0058] Grillet L , Lan P, Li W, Mokkapati G, Schmidt W. 2018. IRON MAN is a ubiquitous family of peptides that control iron transport in plants. Nature Plants 4, 953–963. 30323182 10.1038/s41477-018-0266-y

[CIT0059] Grillet L , Ouerdane L, Flis P, Hoang MT, Isaure MP, Lobinski R, Curie C, Mari S. 2014. Ascorbate efflux as a new strategy for iron reduction and transport in plants. Journal of Biological Chemistry 289, 2515–2525. 24347170 10.1074/jbc.M113.514828 PMC3908387

[CIT0060] Hanna DA , Harvey RM, Martinez-Guzman O, Yuan X, Chandrasekharan B, Raju G, Outten FW, Hamza I, Reddi AR. 2016. Heme dynamics and trafficking factors revealed by genetically encoded fluorescent heme sensors. Proceedings of the National Academy of Sciences, USA 113, 7539–7544. 10.1073/pnas.1523802113 PMC4941510 27247412

[CIT0061] Hanikenne M , Esteves SM, Fanara S, Rouached H. 2021. Coordinated homeostasis of essential mineral nutrients: a focus on iron. Journal of Experimental Botany 72, 2136–2153. 33175167 10.1093/jxb/eraa483

[CIT0062] Hantzis LJ , Kroh GE, Jahn CE, Cantrell M, Peers G, Pilon M, Ravet K. 2018. A program for iron economy during deficiency targets specific Fe proteins. Plant Physiology 176, 596–610. 29150559 10.1104/pp.17.01497 PMC5761800

[CIT0063] Harris WR. 2002. Iron chemistry. In: Templeton DM ed. Molecular and cellular iron transport. New York: Marcel Dekker, 1–48.

[CIT0064] Harris CJ , Scheibe M, Wongpalee SP, et al. 2018. A DNA methylation reader complex that enhances gene transcription. Science 362, 1182–1186. 30523112 10.1126/science.aar7854 PMC6353633

[CIT0065] Haydon MJ , Kawachi M, Wirtz M, Hillmer S, Hell R, Krämer U. 2012. Vacuolar nicotianamine has critical and distinct roles under iron deficiency and for zinc sequestration in *Arabidopsis*. The Plant Cell 24, 724–737. 22374397 10.1105/tpc.111.095042 PMC3315243

[CIT0066] Hernández-Gallardo A K , Missirlis F. 2020. Cellular iron sensing and regulation: nuclear IRP1 extends a classic paradigm. Biochimica et Biophysica Acta - Molecular Cell Research 1867, 118705. 32199885 10.1016/j.bbamcr.2020.118705

[CIT0067] Hider R , Aviles MV, Chen YL, Latunde-Dada GO. 2021. The role of GSH in intracellular iron trafficking. International Journal of Molecular Sciences, 22, 1278. 33525417 10.3390/ijms22031278 PMC7865746

[CIT0068] Hider RC , Kong X. 2010. Chemistry and biology of siderophores. Natural Product Reports 27, 637–657. 20376388 10.1039/b906679a

[CIT0069] Hider RC , Kong XL. 2011. Glutathione: a key component of the cytoplasmic labile iron pool. Biometals 24, 1179–1187. 21769609 10.1007/s10534-011-9476-8

[CIT0070] Hider RC , Kong X. 2013. Iron: effect of overload and deficiency. In: Sigel S, Sigel H, Sigel RKO eds. Interrelations between essential metal ions and human diseases. Dordrecht: Springer, 229–294. 10.1007/978-94-007-7500-8_8 24470094

[CIT0071] Hider RC , Yoshimura E, Khodr H, Von Wirén N. 2004. Competition or complementation: the iron-chelating abilities of nicotianamine and phytosiderophores. New Phytologist 164, 204–208. 33873550 10.1111/j.1469-8137.2004.01209.x

[CIT0072] Hindt MN , Akmakjian GZ, Pivarski KL, Punshon T, Baxter I, Salt DE, Guerinot ML. 2017. *BRUTUS* and its paralogs, *BTS LIKE1* and *BTS LIKE2*, encode important negative regulators of the iron deficiency response in *Arabidopsis thaliana*. Metallomics 9, 876–890. 28620661 10.1039/c7mt00152e PMC5558852

[CIT0073] Hoang MTT , Almeida D, Chay S, Alcon C, Faillie C, Curie C, Mari S. 2021. AtDTX25, a member of the multidrug and toxic compound extrusion family, is a vacuolar ascorbate transporter that controls intracellular iron cycling in Arabidopsis. New Phytologist 231, 1956–1967. 34080200 10.1111/nph.17526

[CIT0074] Hu X , Page MT, Sumida A, Tanaka A, Terry MJ, Tanaka R. 2017. The iron–sulfur cluster biosynthesis protein SUFB is required for chlorophyll synthesis, but not phytochrome signaling. The Plant Journal 89, 1184–1194. 28004871 10.1111/tpj.13455 PMC5347852

[CIT0075] Iñigo S , Durand AN, Ritter A, et al. 2016. Glutaredoxin GRXS17 associates with the cytosolic iron-sulfur cluster assembly pathway. Plant Physiology 172, 858–873. 27503603 10.1104/pp.16.00261 PMC5047072

[CIT0076] Izaguirre-Mayoral ML , Sinclair TR. 2009. Irradiance regulates genotype-specific responses of *Rhizobium*-nodulated soybean to increasing iron and two manganese concentrations in solution culture. Journal of Plant Physiology 166, 807–818. 19108931 10.1016/j.jplph.2008.10.006

[CIT0077] Jain A , Dashner ZS, Connolly EL. 2019. Mitochondrial iron transporters (MIT1 and MIT2) are essential for iron homeostasis and embryogenesis in *Arabidopsis thaliana*. Frontiers in Plant Science 10, 1449. 31850005 10.3389/fpls.2019.01449 PMC6889801

[CIT0078] Jeong J , Cohu C, Kerkeb L, Pilon M, Connolly EL, Guerinot ML. 2008. Chloroplast Fe(III) chelate reductase activity is essential for seedling viability under iron limiting conditions. Proceedings of the National Academy of Sciences, USA 105, 10619–10624. 10.1073/pnas.0708367105 PMC2492473 18647837

[CIT0079] Kailasam S , Wang Y, Lo JC, Chang HF, Yeh KC. 2018. *S*-nitrosoglutathione works downstream of nitric oxide to mediate iron-deficiency signaling in Arabidopsis. The Plant Journal 94, 157–168. 29396986 10.1111/tpj.13850

[CIT0080] Karmi O , Marjault HB, Pesce L, Carloni P, Jose’N O, Jennings PA, Mittler R, Nechushtai R. 2018. The unique fold and lability of the [2Fe–2S] clusters of NEET proteins mediate their key functions in health and disease. Journal of Biological Inorganic Chemistry 23, 599–612. 29435647 10.1007/s00775-018-1538-8 PMC6006223

[CIT0081] Kato Y , Watanabe H, Noguchi T. 2021. Spectroelectrochemical study on the mechanism of the pH dependence of the redox potential of the non-heme iron in photosystem II. Biochemistry 60, 2170–2178. 34181388 10.1021/acs.biochem.1c00341

[CIT0082] Khan MA , Castro-Guerrero NA, McInturf SA, et al. 2018. Changes in iron availability in Arabidopsis are rapidly sensed in the leaf vasculature and impaired sensing leads to opposite transcriptional programs in leaves and roots. Plant, Cell and Environment 41, 2263–2276. 10.1111/pce.13192 29520929

[CIT0083] Kim LJ , Tsuyuki KM, Hu F, et al. 2021. Ferroportin 3 is a dual-targeted mitochondrial/chloroplast iron exporter necessary for iron homeostasis in Arabidopsis. The Plant Journal 107, 215–236. 33884692 10.1111/tpj.15286 PMC8316378

[CIT0084] Kim SA , Punshon T, Lanzirotti A, Li L, Alonso JM, Ecker JR, Kaplan J, Guerinot ML. 2006. Localization of iron in *Arabidopsis* seed requires the vacuolar membrane transporter VIT1. Science 314, 1295–1298. 17082420 10.1126/science.1132563

[CIT0085] Klatte M , Schuler M, Wirtz M, Fink-Straube C, Hell R, Bauer P. 2009. The analysis of Arabidopsis nicotianamine synthase mutants reveals functions for nicotianamine in seed iron loading and iron deficiency responses. Plant Physiology 150, 257–271. 19304929 10.1104/pp.109.136374 PMC2675739

[CIT0086] Kobayashi T. 2019. Understanding the complexity of iron sensing and signaling cascades in plants. Plant and Cell Physiology 60, 1440–1446. 30796837 10.1093/pcp/pcz038

[CIT0087] Kobayashi T , Nagasaka S, Senoura T, Itai RN, Nakanishi H, Nishizawa NK. 2013. Iron-binding haemerythrin RING ubiquitin ligases regulate plant iron responses and accumulation. Nature Communications 4, 1–12. 10.1038/ncomms3792 PMC3905729 24253678

[CIT0088] Kruse I , Maclean AE, Hill L, Balk J. 2018. Genetic dissection of cyclic pyranopterin monophosphate biosynthesis in plant mitochondria. Biochemical Journal 475, 495–509. 29247140 10.1042/BCJ20170559 PMC5791162

[CIT0089] Kumar C , Igbaria A, D’Autreaux B, Planson AG, Junot C, Godat E, Bachhawat AK, Delaunay-Moisan A, Toledano MB. 2011. Glutathione revisited: a vital function in iron metabolism and ancillary role in thiol-redox control. EMBO Journal 30, 2044–2056. 21478822 10.1038/emboj.2011.105 PMC3098478

[CIT0090] Kumar RK , Chu HH, Abundis C, Vasques K, Rodriguez DC, Chia JC, Huang R, Vatamaniuk OK, Walker EL. 2017. Iron-nicotianamine transporters are required for proper long distance iron signaling. Plant Physiology 175, 1254–1268. 28894019 10.1104/pp.17.00821 PMC5664466

[CIT0091] Landry AP , Duan X, Huang H, Ding H. 2011. Iron–sulfur proteins are the major source of protein-bound dinitrosyl iron complexes formed in *Escherichia coli* cells under nitric oxide stress. Free Radical Biology and Medicine 50, 1582–1590. 21420489 10.1016/j.freeradbiomed.2011.03.005 PMC3090472

[CIT0092] Lanquar V , Lelièvre F, Bolte S, et al. 2005. Mobilization of vacuolar iron by AtNRAMP3 and AtNRAMP4 is essential for seed germination on low iron. The EMBO Journal 24, 4041–4051. 16270029 10.1038/sj.emboj.7600864 PMC1356305

[CIT0093] Lanquar V , Ramos MS, Lelièvre F, Barbier-Brygoo H, Krieger-Liszkay A, Krämer U, Thomine S. 2010. Export of vacuolar manganese by AtNRAMP3 and AtNRAMP4 is required for optimal photosynthesis and growth under manganese deficiency. Plant Physiology 152, 1986–1999. 20181755 10.1104/pp.109.150946 PMC2850043

[CIT0094] Larbi A , Morales F, Abadía A, Abadía J. 2010. Changes in iron and organic acid concentrations in xylem sap and apoplastic fluid of iron-deficient *Beta vulgaris* plants in response to iron resupply. Journal of Plant Physiology 167, 255–260. 19854536 10.1016/j.jplph.2009.09.007

[CIT0095] Larbi A , Morales F, López-Millán A, Gogorcena Y, Abadía A, Moog P, Abadía J. 2001. Technical advance: Reduction of Fe(III)-chelates by mesophyll leaf disks of sugar beet. Multi-component origin and effects of Fe deficiency. Plant and Cell Physiology 42, 94–105. 11158448 10.1093/pcp/pce012

[CIT0096] Lee S , Ricachenevsky FK, Punshon T. 2021. Functional overlap of two major facilitator superfamily transporter, ZIF1, and ZIFL1 in zinc and iron homeostasis. Biochemical and Biophysical Research Communications 560, 7–13. 33964505 10.1016/j.bbrc.2021.04.120

[CIT0097] Lewandowska H , Kalinowska M, Brzóska K, Wójciuk K, Wójciuk G, Kruszewski M. 2011. Nitrosyl iron complexes—synthesis, structure and biology. Dalton Transactions 40, 8273–8289. 21643591 10.1039/c0dt01244k

[CIT0098] Li J , Cowan JA. 2015. Glutathione-coordinated [2Fe–2S] cluster: a viable physiological substrate for mitochondrial ABCB7 transport. Chemical Communications 51, 2253–2255. 25556595 10.1039/c4cc09175b PMC4522903

[CIT0099] Li L , Li L. 2016. Recent advances in multinuclear metal nitrosyl complexes. Coordination Chemistry Reviews 306, 678–700. 26744544 10.1016/j.ccr.2015.03.026 PMC4698915

[CIT0100] Li L , Ye L, Kong Q, Shou H. 2019. A vacuolar membrane ferric-chelate reductase, OsFRO1, alleviates Fe toxicity in rice (*Oryza sativa* L.). Frontiers in Plant Science 10, 700. 31214220 10.3389/fpls.2019.00700 PMC6558154

[CIT0101] Li LY , Cai QY, Yu DS, Guo CH. 2011. Overexpression of *AtFRO6* in transgenic tobacco enhances ferric chelate reductase activity in leaves and increases tolerance to iron-deficiency chlorosis. Molecular Biology Reports 38, 3605–3613. 21104018 10.1007/s11033-010-0472-9

[CIT0102] Lide DR , ed. 2004. CRC handbook of chemistry and physics (84th edition). Boca Raton: CRC Press.

[CIT0103] Lill R , Freibert SA. 2020. Mechanisms of mitochondrial iron-sulfur protein biogenesis. Annual Review of Biochemistry 89, 471–499. 10.1146/annurev-biochem-013118-111540 31935115

[CIT0104] Liu DH , Adler K, Stephan UW. 1998. Iron-containing particles accumulate in organelles and vacuoles of leaf and root cells in the nicotianamine-free tomato mutant *chloronerva*. Protoplasma 201, 213–220.

[CIT0105] Long TA , Tsukagoshi H, Busch W, Lahner B, Salt DE, Benfey PN. 2010. The bHLH transcription factor POPEYE regulates response to iron deficiency in *Arabidopsis* roots. The Plant Cell 22, 2219–2236. 20675571 10.1105/tpc.110.074096 PMC2929094

[CIT0106] López-Millán A-F , Morales F, Abadía A, Abadía J. 2000. Effects of iron deficiency on the composition of the leaf apoplastic fluid and xylem sap in sugar beet. Implications for iron and carbon transport. Plant Physiology 124, 873–884. 11027735 10.1104/pp.124.2.873 PMC59191

[CIT0107] Lu Y. 2018. Assembly and transfer of iron–sulfur clusters in the plastid. Frontiers in Plant Science 9, 336. 29662496 10.3389/fpls.2018.00336 PMC5890173

[CIT0108] Maliandi MV , Busi MV, Turowski VR, Leaden L, Araya A, Gomez-Casati DF. 2011. The mitochondrial protein frataxin is essential for heme biosynthesis in plants. FEBS Journal 278, 470–481. 21166997 10.1111/j.1742-4658.2010.07968.x

[CIT0109] Mari S , Bailly C, Thomine S. 2020. Handing off iron to the next generation: how does it get into seeds and what for? Biochemical Journal 477, 259–274. 31950999 10.1042/BCJ20190188

[CIT0110] Martell AE , Hancock RD. 1996. Metal complexes in aqueous solutions. New York: Plenum Press.

[CIT0111] Martell AE , Smith M. 1977. Other organic ligands. New York: Plenum Press.

[CIT0112] Martínez-Ballesta MDC , Egea-Gilabert C, Conesa E, Ochoa J, Vicente MJ, Franco JA, Bañon S, Martínez JJ, Fernández JA. 2020. The importance of ion homeostasis and nutrient status in seed development and germination. Agronomy 10, 504.

[CIT0113] Martins L , Knuesting J, Bariat L, et al. 2020. Redox modification of the iron-sulfur glutaredoxin GRXS17 activates holdase activity and protects plants from heat stress. Plant Physiology 184, 676–692. 32826321 10.1104/pp.20.00906 PMC7536686

[CIT0114] Marty L , Bausewein D, Müller C, et al. 2019. Arabidopsis glutathione reductase 2 is indispensable in plastids, while mitochondrial glutathione is safeguarded by additional reduction and transport systems. New Phytologist 224, 1569–1584. 31372999 10.1111/nph.16086

[CIT0115] Meyer A J , May MJ, Fricker M. 2001. Quantitative *in vivo* measurement of glutathione in *Arabidopsis* cells. The Plant Journal 27, 67–78. 11489184 10.1046/j.1365-313x.2001.01071.x

[CIT0116] Miller CN , Busch W. 2021. Using natural variation to understand plant responses to iron availability. Journal of Experimental Botany 72, 2154–2164. 33458759 10.1093/jxb/erab012 PMC7966951

[CIT0117] Monsant AC , Kappen P, Wang Y, Pigram PJ, Baker AJM, Tang C. 2011. In vivo speciation of zinc in *Noccaea caerulescens* in response to nitrogen form and zinc exposure. Plant and Soil 348, 167.

[CIT0118] Moseler A , Aller I, Wagner S, et al. 2015. The mitochondrial monothiol glutaredoxin S15 is essential for iron-sulfur protein maturation in *Arabidopsis thaliana*. Proceedings of the National Academy of Sciences, USA 112, 13735–13740. 10.1073/pnas.1510835112 PMC4640787 26483494

[CIT0119] Mukherjee I , Campbell NH, Ash JS, Connolly EL. 2006. Expression profiling of the Arabidopsis ferric chelate reductase (*FRO*) gene family reveals differential regulation by iron and copper. Planta 223, 1178–1190. 16362328 10.1007/s00425-005-0165-0

[CIT0120] Murgia I , Vigani G. 2015. Analysis of *Arabidopsis thaliana atfer4-1*, *atfh* and *atfer4-1*/*atfh* mutants uncovers frataxin and ferritin contributions to leaf ionome homeostasis. Plant Physiology and Biochemistry 94, 65–72. 26042547 10.1016/j.plaphy.2015.05.011

[CIT0121] Mühlenhoff U , Braymer JJ, Christ S, Rietzschel N, Uzarska MA, Weiler BD, Lill R. 2020. Glutaredoxins and iron-sulfur protein biogenesis at the interface of redox biology and iron metabolism. Biological Chemistry 401, 1407–1428. 33031050 10.1515/hsz-2020-0237

[CIT0122] Murphy JM , Powell BA, Brumaghim JL. 2020. Stability constants of bio-relevant, redox-active metals with amino acids: the challenges of weakly binding ligands. Coordination Chemistry Reviews 412, 213253–213273.

[CIT0123] Müller B , Kovács K, Pham HD, et al. 2019. Chloroplasts preferentially take up ferric–citrate over iron–nicotianamine complexes in *Brassica napus*. Planta 249, 751–763. 30382344 10.1007/s00425-018-3037-0

[CIT0124] Nechushtai R , Conlan AR, Harir Y, et al. 2012. Characterization of *Arabidopsis* NEET reveals an ancient role for NEET proteins in iron metabolism. The Plant Cell 24, 2139–2154. 22562611 10.1105/tpc.112.097634 PMC3442592

[CIT0125] Nechushtai R , Karmi O, Zuo K, Marjault HB, Darash-Yahana M, Sohn YS, King SD, Zandalinas SI, Carloni P, Mittler R. 2020. The balancing act of NEET proteins: iron, ROS, calcium and metabolism. Biochimica et Biophysica Acta - Molecular Cell Research 1867, 118805. 32745723 10.1016/j.bbamcr.2020.118805

[CIT0126] O’Keeffe R , Latunde-Dada GO, Chen YL, Kong XL, Cilibrizzi A, Hider RC. 2021. Glutathione and the intracellular labile heme pool. Biometals 34, 221–228. 33301081 10.1007/s10534-020-00274-w PMC7940311

[CIT0127] O’Neil MJ. 2013. The Merck index - an encyclopedia of chemicals, drugs, and biologicals (15th edition). Cambridge: Royal Society of Chemistry.

[CIT0128] Park EY , Tsuyuki KM, Parsons EM, Jeong J. 2020. PRC2-mediated H3K27me3 modulates shoot iron homeostasis in *Arabidopsis thaliana*. Plant Signaling and Behavior 15, 1784549. 32594838 10.1080/15592324.2020.1784549 PMC8550290

[CIT0129] Petit JM , van Wuytswinkel O, Briat JF, Lobréaux S. 2001. Characterization of an iron-dependent regulatory sequence involved in the transcriptional control of *AtFer1* and *ZmFer1* plant ferritin genes by iron. Journal of Biological Chemistry 276, 5584–5590. 11092880 10.1074/jbc.M005903200

[CIT0130] Pham HD , Pólya S, Müller B, et al. 2020. The developmental and iron nutritional pattern of PIC1 and NiCo does not support their interdependent and exclusive collaboration in chloroplast iron transport in *Brassica napus*. Planta 251, 96. 32297017 10.1007/s00425-020-03388-0 PMC7214486

[CIT0131] Pottier M , Dumont J, Masclaux-Daubresse C, Thomine S. 2019. Autophagy is essential for optimal translocation of iron to seeds in Arabidopsis. Journal of Experimental Botany 70, 859–869. 30395253 10.1093/jxb/ery388 PMC6363094

[CIT0132] Prasetyo E , Anderson C, Nurjaman F, Al Muttaqii M, Handoko AS, Bahfie F, Mufakhir FR. 2020. Monosodium glutamate as selective lixiviant for alkaline leaching of zinc and copper from electric arc furnace dust. Metals 10, 644.

[CIT0133] Przybyla-Toscano J , Christ L, Keech O, Rouhier N. 2021. Iron–sulfur proteins in plant mitochondria: roles and maturation. Journal of Experimental Botany 72, 2014–2044. 33301571 10.1093/jxb/eraa578

[CIT0134] Przybyla-Toscano J , Roland M, Gaymard F, Couturier J, Rouhier N. 2018. Roles and maturation of iron–sulfur proteins in plastids. Journal of Biological Inorganic Chemistry 23, 545–566. 29349662 10.1007/s00775-018-1532-1 PMC6006212

[CIT0135] Qi W , Li J, Chain CY, Pasquevich GA, Pasquevich AF, Cowan JA. 2012. Glutathione complexed Fe–S centers. Journal of the American Chemical Society 134, 10745–10748. 22687047 10.1021/ja302186j PMC3401418

[CIT0136] Ramirez L , Simontacchi M, Murgia I, Zabaleta E, Lamattina L. 2011. Nitric oxide, nitrosyl iron complexes, ferritin and frataxin: a well-equipped team to preserve plant iron homeostasis. Plant Science 181, 582–592. 21893255 10.1016/j.plantsci.2011.04.006

[CIT0137] Ravet K , Touraine B, Boucherez J, Briat JF, Gaymard F, Cellier F. 2009. Ferritins control interaction between iron homeostasis and oxidative stress in Arabidopsis. The Plant Journal 57, 400–412. 18826427 10.1111/j.1365-313X.2008.03698.x

[CIT0138] Rellán-Álvarez R , Giner-Martínez-Sierra J, Orduna J, Orera I, Rodríguez-Castrillón JA, García-Alonso JI, Abadía J, Álvarez-Fernández A. 2010. Identification of a tri-iron (III), tri-citrate complex in the xylem sap of iron-deficient tomato resupplied with iron: new insights into plant iron long-distance transport. Plant and Cell Physiology 51, 91–102. 19942594 10.1093/pcp/pcp170

[CIT0139] Rey P , Taupin-Broggini M, Couturier J, Vignols F, Rouhier N. 2019. Is there a role for glutaredoxins and BOLAs in the perception of the cellular iron status in plants? Frontiers in Plant Science 10, 712. 31231405 10.3389/fpls.2019.00712 PMC6558291

[CIT0140] Riaz N , Guerinot ML. 2021. All together now: regulation of the iron deficiency response. Journal of Experimental Botany 72, 2045–2055. 33449088 10.1093/jxb/erab003 PMC7966950

[CIT0141] Rodríguez-Celma J , Chou H, Kobayashi T, Long TA, Balk J. 2019. Hemerythrin E3 ubiquitin ligases as negative regulators of iron homeostasis in plants. Frontiers in Plant Science 10, 98. 30815004 10.3389/fpls.2019.00098 PMC6381054

[CIT0142] Rodríguez-Celma J , Pan I, Li WD, Lan PD, Buckhout TJ, Schmidt W. 2013. The transcriptional response of *Arabidopsis* leaves to Fe deficiency. Frontiers in Plant Science 4, 276. 23888164 10.3389/fpls.2013.00276 PMC3719017

[CIT0143] Roret T , Tsan P, Couturier J, Zhang B, Johnson MK, Rouhier N, Didierjean C. 2014. Structural and spectroscopic insights into BolA-glutaredoxin complexes. Journal of Biological Chemistry 289, 24588–24598. 25012657 10.1074/jbc.M114.572701 PMC4148882

[CIT0144] Roschzttardtz H , Conéjéro G, Curie C, Mari S. 2009. Identification of the endodermal vacuole as the iron storage compartment in the Arabidopsis embryo. Plant Physiology 151, 1329–1338. 19726572 10.1104/pp.109.144444 PMC2773051

[CIT0145] Roschzttardtz H , Conéjéro G, Divol F, Alcon C, Verdeil JL, Curie C, Mari S. 2013. New insights into Fe localization in plant tissues. Frontiers in Plant Science 4, 350. 24046774 10.3389/fpls.2013.00350 PMC3764369

[CIT0146] Roschzttardtz H , Séguéla-Arnaud M, Briat JF, Vert G, Curie C. 2011. The FRD3 citrate effluxer promotes iron nutrition between symplastically disconnected tissues throughout *Arabidopsis* development. The Plant Cell 23, 2725–2737. 21742986 10.1105/tpc.111.088088 PMC3226209

[CIT0147] Sági-Kazár M , Zelenyánszki H, Müller B, et al. 2021. Supraoptimal iron nutrition of *Brassica napus* plants suppresses the iron uptake of chloroplasts by down-regulating chloroplast ferric chelate reductase. Frontiers in Plant Science 12, 748. 10.3389/fpls.2021.658987 PMC8172622 34093616

[CIT0148] Sahini VE , Dumitrescu M, Volanschi E, Birla L, Diaconu C. 1996. Spectral and interferometrical study of the interaction of haemin with glutathione. Biophysical Chemistry 58, 245–253. 8820409 10.1016/0301-4622(95)00110-7

[CIT0149] Schaedler TA , Thornton JD, Kruse I, Schwarzländer M, Meyer AJ, Van Veen HW, Balk J. 2014. A conserved mitochondrial ATP-binding cassette transporter exports glutathione polysulfide for cytosolic metal cofactor assembly. Journal of Biological Chemistry 289, 23264–23274. 25006243 10.1074/jbc.M114.553438 PMC4156053

[CIT0150] Schuler M , Rellán-Álvarez R, Fink-Straube C, Abadía J, Bauer P. 2012. Nicotianamine functions in the phloem-based transport of iron to sink organs, in pollen development and pollen tube growth in *Arabidopsis*. The Plant Cell 24, 2380–2400. 22706286 10.1105/tpc.112.099077 PMC3406910

[CIT0151] Sebastian A , Prasad MNV. 2018. Exogenous citrate and malate alleviate cadmium stress in *Oryza sativa* L.: probing role of cadmium localization and iron nutrition. Ecotoxicology and Environmental Safety 166, 215–222. 30269017 10.1016/j.ecoenv.2018.09.084

[CIT0152] Seckback J. 1982. Ferreting out the secrets of plant ferritin - a review. Journal of Plant Nutrition 5, 369–394.

[CIT0153] Senoura T , Kobayashi T, An G, Nakanishi H, Nishizawa NK. 2020. Defects in the rice aconitase-encoding *OsACO1* gene alter iron homeostasis. Plant Molecular Biology 104, 629–645. 32909184 10.1007/s11103-020-01065-0

[CIT0154] Séré D , Martin A. 2020. Epigenetic regulation: another layer in plant nutrition. Plant Signaling and Behavior 15, 1–6. 10.1080/15592324.2019.1686236 PMC7012064 31674259

[CIT0155] Seregin IV , Kozhevnikova AD. 2020. Low-molecular-weight ligands in plants: role in metal homeostasis and hyperaccumulation. Photosynthesis Research 150, 51–96. 32653983 10.1007/s11120-020-00768-1

[CIT0156] Shafiq S , Ali A, Sajjad Y, Zeb Q, Shahzad M, Khan AR, Nazir R, Widemann E. 2020. The interplay between toxic and essential metals for their uptake and translocation is likely governed by DNA methylation and histone deacetylation in maize. International Journal of Molecular Sciences 21, 6959. 10.3390/ijms21186959 PMC7555519 32971934

[CIT0157] Shi R , Weber G, Köster J, Reza-Hajirezaei M, Zou C, Zhang F, von Wirén N. 2012. Senescence-induced iron mobilization in source leaves of barley (*Hordeum vulgare*) plants. New Phytologist 195, 372–383. 22591276 10.1111/j.1469-8137.2012.04165.x

[CIT0158] Shee R , Ghosh S, Khan P, Sahid S, Roy C, Shee D, Paul S, Datta R. 2021. Glutathione regulates subcellular iron homeostasis via transcriptional activation of iron responsive genes in *Arabidopsis*. bioRxiv doi: 10.1101/2021.05.09.443283. [Preprint]. 10.1111/pce.14331 35394650

[CIT0159] Shimizu T , Yasuda R, Mukai Y, Tanoue R, Shimada T, Imamura S, Tanaka K, Watanabe S, Masuda T. 2020. Proteomic analysis of haem-binding protein from *Arabidopsis thaliana* and *Cyanidioschyzon merolae*. Philosophical Transactions of the Royal Society B 375, 20190488. 10.1098/rstb.2019.0488 PMC7209954 32362261

[CIT0160] Shviro Y , Shaklai N. 1987. Glutathione as a scavenger of free hemin. A mechanism of preventing red cell membrane damage. Biochemical Pharmacology 36, 3801–3807. 3689422 10.1016/0006-2952(87)90441-2

[CIT0161] Silva AM , Kong X, Parkin MC, Cammack R, Hider RC. 2009. Iron(III) citrate speciation in aqueous solution. Dalton Transactions 40, 8616–8625. 10.1039/b910970f 19809738

[CIT0162] Silverstein TP , Heller ST. 2017. pK_a_ values in the undergraduate curriculum: what is the real pK_a_ of water? Journal of Chemical Education 94, 690–695.

[CIT0163] Solti Á , Kovács K, Basa B, Vértes A, Sárvári E, Fodor F. 2012. Uptake and incorporation of iron in sugar beet chloroplasts. Plant Physiology and Biochemistry 52, 91–97. 22305071 10.1016/j.plaphy.2011.11.010

[CIT0164] Solti Á , Müller B, Czech V, Sárvári É, Fodor F. 2014. Functional characterization of the chloroplast ferric chelate oxidoreductase enzyme. New Phytologist 202, 920–928. 24506824 10.1111/nph.12715

[CIT0165] Solymosi K , Martinez K, Kristóf Z, Sundqvist C, Böddi B. 2004. Plastid differentiation and chlorophyll biosynthesis in different leaf layers of white cabbage (*Brassica oleracea* cv. *capitata*). Physiologia Plantarum 121, 520–529.

[CIT0166] Solymosi K , Morandi D, Bóka K, Böddi B, Schoefs B. 2012. High biological variability of plastids, photosynthetic pigments and pigment forms of leaf primordia in buds. Planta 235, 1035–1104. 22160501 10.1007/s00425-011-1559-9

[CIT0167] Solymosi K , Mysliwa-Kurdziel B. 2021. The role of membranes and lipid-protein interactions in the Mg-branch of tetrapyrrole biosynthesis. Frontiers in Plant Science 28, 663309. 10.3389/fpls.2021.663309 PMC8113382 33995458

[CIT0168] Su LW , Chang SH, Li MY, Huang HY, Jane WN, Yang JY. 2013. Purification and biochemical characterization of *Arabidopsis* At-NEET, an ancient iron-sulfur protein, reveals a conserved cleavage motif for subcellular localization. Plant Science 213, 46–54. 24157207 10.1016/j.plantsci.2013.09.001

[CIT0169] Sun S , Zhu J, Guo R, Whelan J, Shou H. 2021. DNA methylation is involved in acclimation to iron deficiency in rice (*Oryza sativa*). The Plant Journal 107, 727–73. 33977637 10.1111/tpj.15318

[CIT0170] Suzuki K , Itai R, Suzuki K, Nakanishi H, Nishizawa NK, Yoshimura E, Mori S. 1998. Formate dehydrogenase, an enzyme of anaerobic metabolism, is induced by iron deficiency in barley roots. Plant Physiology 116, 725–732. 9489019 10.1104/pp.116.2.725 PMC35132

[CIT0171] Sylvestre-Gonon E , Schwartz M, Girardet J-M, Hecker A, Rouhier N. 2020. Is there a role for tau glutathione transferases in tetrapyrrole metabolism and retrograde signalling in plants? Philosophical Transactions of the Royal Society B 375, 20190404. 10.1098/rstb.2019.0404 PMC7209958 32362257

[CIT0172] Talib E , Outten CE. 2021. Iron-sulfur cluster biogenesis, trafficking, and signaling: roles for CGFS glutaredoxins and BolA proteins. Biochimica et Biophysica Acta - Molecular Cell Research 1868, 118847. 32910989 10.1016/j.bbamcr.2020.118847 PMC7837452

[CIT0173] Tarantino D , Petit JM, Lobreaux S, Briat JF, Soave C, Murgia I. 2003. Differential involvement of the IDRS *cis*-element in the developmental and environmental regulation of the *AtFer1* ferritin gene from *Arabidopsis*. Planta 217, 709–716. 12728319 10.1007/s00425-003-1038-z

[CIT0174] Terry N , Abadía J. 1986. Function of iron in chloroplasts. Journal of Plant Nutrition 9, 609–646.

[CIT0175] Tewari D , Sah AN, Bawari S, Nabavi SF, Dehpour AR, Shirooie S, Braidy N, Fiebich BL, Vacca RA, Nabavi SM. 2021. Role of nitric oxide in neurodegeneration: function, regulation, and inhibition. Current Neuropharmacology 19, 114–126. 32348225 10.2174/1570159X18666200429001549 PMC8033982

[CIT0176] Theil EC. 2013. Ferritin: the protein nanocage and iron biomineral in health and in disease. Inorganic Chemistry 52, 12223–12233. 24102308 10.1021/ic400484n PMC3882016

[CIT0177] Theil EC , Briat JF. 2004. Plant ferritin and non-heme iron nutrition in humans. HarvestPlus Technical Monograph 1. Washington DC: International Food Policy Research Institute and International Center for Tropical Agriculture, 1–22.

[CIT0178] Timperio AM , D’Amici GM, Barta C, Loreto F, Zolla L. 2007. Proteomics, pigment composition, and organization of thylakoid membranes in iron-deficient spinach leaves. Journal of Experimental Botany 58, 3695–3710. 17928371 10.1093/jxb/erm219

[CIT0179] Tissot N , Robe K, Gao F, et al. 2019. Transcriptional integration of the responses to iron availability in Arabidopsis by the bHLH factor ILR3. New Phytologist 223, 1433–1446. 30773647 10.1111/nph.15753

[CIT0180] Tripathy BC , Sherameti I, Oelmüller R. 2010. Siroheme: an essential component for life on earth. Plant Signaling and Behavior 5, 14–20. 20592802 10.4161/psb.5.1.10173 PMC2835951

[CIT0181] Trnka D , Engelke AD, Gellert M, et al. 2020. Molecular basis for the distinct functions of redox-active and FeS-transfering glutaredoxins. Nature Communications 11, 1–12. 10.1038/s41467-020-17323-0 PMC7351949 32651396

[CIT0182] Turowski VR , Aknin C, Maliandi MV, Buchensky C, Leaden L, Peralta DA, Busi MV, Araya A, Gomez-Casati DF. 2015. Frataxin is localized to both the chloroplast and mitochondrion and is involved in chloroplast Fe–S protein function in *Arabidopsis*. PLoS One 10, e0141443. 26517126 10.1371/journal.pone.0141443 PMC4636843

[CIT0183] Vanin AF. 2009. Dinitrosyl iron complexes with thiolate ligands: physico-chemistry, biochemistry and physiology. Nitric Oxide 21, 1–13. 19366636 10.1016/j.niox.2009.03.005

[CIT0184] Vigani G , Bashir K, Ishimaru Y, Lehmann M, Casiraghi FM, Nakanishi H, Seki M, Geigenberger P, Zocchi G, Nishizawa NK. 2016. Knocking down mitochondrial iron transporter (MIT) reprograms primary and secondary metabolism in rice plants. Journal of Experimental Botany 67, 1357–1368. 26685186 10.1093/jxb/erv531 PMC4762380

[CIT0185] Vigani G , Di Silvestre D, Agresta AM, Donnini S, Mauri P, Gehl C, Bittner F, Murgia I. 2017. Molybdenum and iron mutually impact their homeostasis in cucumber (*Cucumis sativus*) plants. New Phytologist 213, 1222–1241. 27735062 10.1111/nph.14214

[CIT0186] Vigani G , Hanikenne M. 2018. Metal homeostasis in plant mitochondria. In: Logan DC, ed. Plant mitochondria (2nd edition), Annual Plant Reviews, Vol. 50. Chichester: Wiley Blackwell, 111–142.

[CIT0187] Vigani G , Solti Á, Thomine S, Philippar K. 2019. Essential and detrimental — an update on intracellular iron trafficking and homeostasis. Plant and Cell Physiology 60, 1420–1439. 31093670 10.1093/pcp/pcz091

[CIT0188] Voith von Voithenberg L , Park J, Stübe R, Lux C, Lee Y, Philippar K. 2019. A novel prokaryote-type ECF/ABC transporter module in chloroplast metal homeostasis. Frontiers in Plant Science 10, 1264. 31736987 10.3389/fpls.2019.01264 PMC6828968

[CIT0189] von Wirén N , Klair S, Bansal S, Briat J-F, Khodr H, Shioiri T, Leigh RA, Hider RC. 1999. Nicotianamine chelates both Fe^III^ and Fe^II^. Implications for metal transport in plants. Plant Physiology 119, 1107–1114. 10069850 10.1104/pp.119.3.1107 PMC32093

[CIT0190] Wang J , Chen Q, Wu W, Chen Y, Zhou Y, Guo G, Chen M. 2021. Genome-wide analysis of long non-coding RNAs responsive to multiple nutrient stresses in *Arabidopsis thaliana*. Functional and Integrative Genomics 21, 17–30. 33130916 10.1007/s10142-020-00758-5

[CIT0191] Wang F , Minakhina S, Tran H, Changela N, Kramer J, Steward R. 2018. Tet protein function during Drosophila development. PLoS One 13, e0190367. 29324752 10.1371/journal.pone.0190367 PMC5764297

[CIT0192] Waters BM , Chu HH, DiDonato RJ, Roberts LA, Eisley RB, Lahner B, Salt DE, Walker EL. 2006. Mutations in Arabidopsis *Yellow Stripe-Like1* and *Yellow Stripe-Like3* reveal their roles in metal ion homeostasis and loading of metal ions in seeds. Plant Physiology 141, 1446–1458. 16815956 10.1104/pp.106.082586 PMC1533956

[CIT0193] Weiler BD , Brück MC, Kothe I, Bill E, Lill R, Mühlenhoff U. 2020. Mitochondrial [4Fe–4S] protein assembly involves reductive [2Fe–2S] cluster fusion on ISCA1–ISCA2 by electron flow from ferredoxin FDX2. Proceedings of the National Academy of Sciences, USA 117, 20555–20565. 10.1073/pnas.2003982117 PMC7456137 32817474

[CIT0194] Wen D , Sun S, Yang W, Zhang L, Liu S, Gong B, Shi Q. 2019. Overexpression of *S*-nitrosoglutathione reductase alleviated iron-deficiency stress by regulating iron distribution and redox homeostasis. Journal of Plant Physiology 237, 1–11. 30999072 10.1016/j.jplph.2019.03.007

[CIT0195] Xing J , Wang T, Liu Z, et al. 2015. GENERAL CONTROL NONREPRESSED PROTEIN5-mediated histone acetylation of *FERRIC REDUCTASE DEFECTIVE3* contributes to iron homeostasis in Arabidopsis. Plant Physiology 168, 1309–1320. 26002909 10.1104/pp.15.00397 PMC4528745

[CIT0196] Zandalinas SI , Song L, Sengupta S, et al. 2020. Expression of a dominant-negative AtNEET-H89C protein disrupts iron–sulfur metabolism and iron homeostasis in Arabidopsis. The Plant Journal 101, 1152–1169. 31642128 10.1111/tpj.14581

[CIT0197] Zechmann B , Stumpe M, Mauch F. 2011. Immunocytochemical determination of the subcellular distribution of ascorbate in plants. Planta 233, 1–12. 20872269 10.1007/s00425-010-1275-x PMC3015205

[CIT0198] Zhang C , Römheld V, Marschner H. 1995. Retranslocation of iron from primary leaves of bean plants grown under iron deficiency. Journal of Plant Physiology 146, 268–272.

[CIT0199] Zhang H , Lang Z, Zhu JK. 2018. Dynamics and function of DNA methylation in plants. Nature Reviews Molecular Cell Biology 19, 489–506. 29784956 10.1038/s41580-018-0016-z

[CIT0200] Zhang J , Bai Z, Ouyang M, et al. 2021. The DnaJ proteins DJA6 and DJA5 are essential for chloroplast iron–sulfur cluster biogenesis. The EMBO Journal 40, e106742. 33855718 10.15252/embj.2020106742 PMC8246258

[CIT0201] Zhang Y , Xu YH, Yi HY, Gong JM. 2012. Vacuolar membrane transporters OsVIT1 and OsVIT2 modulate iron translocation between flag leaves and seeds in rice. The Plant Journal 72, 400–410. 22731699 10.1111/j.1365-313X.2012.05088.x

[CIT0202] Zhao G. 2010. Phytoferritin and its implications for human health and nutrition. Biochimica et Biophysica Acta - General Subjects 1800, 815–823. 10.1016/j.bbagen.2010.01.009 20100546

